# Copper-Doped Chromium Oxide Delafossite: Sputtering Process, Material Properties, and Device Applications: A Detailed Review

**DOI:** 10.1021/acsomega.5c01245

**Published:** 2025-11-19

**Authors:** Balaji Gururajan, Deveshwar Sasikumar, Done Rinshun Paul, Wei - Sheng Liu, Bhavya Kondapavuluri, Parthasarathy Srinivasan, Sridharan Madanagurusamy, Jenifer K

**Affiliations:** † Department of Electrical Engineering, 34895 Yuan Ze University, Zhongli, Taoyuan 32003, Taiwan; ‡ Division of Physical Sciences, 121735 Karunya Institute of Technology and Sciences, Coimbatore 641114, India; § Department of Electronics and Communication Engineering, Amrita School of Engineering, Chennai 601103, Amrita Vishwa Vidyapeetham, India; ∥ School of Arts, Sciences, Humanities & Education and Centre for Nanotechnology and Advanced Biomaterials, SASHE, SASTRA Deemed to be University, Thanjavur 613401, Tamil Nadu, India; ⊥ Advanced materials and devices laboratory, 374903 PSG Institute of Advanced Studies, Peelamedu, Coimbatore 641004, India

## Abstract

Transparent conducting oxides (TCOs) are essential for modern optoelectronic technologies, providing a perfect combination of great optical transparency and superior electrical conductivity. Although n-type transparent conductive oxides dominate in the field, the advancement of p-type TCOs is crucial for the realization of entirely transparent electrical devices. Delafossite-based Copper-doped chromium oxide (CuCrO_2_) is a notable p-type TCO, exhibiting significant optical transparency, electrical conductivity, and magnetic adaptability. Its abundant and nontoxic characteristics further augment its attractiveness for sustainable energy and optoelectronic applications. This review examines the fabrication and optimization of sputtered CuCrO_2_ thin films, highlighting deposition procedures, post-treatment methods, and their effects on structural, morphological, electrical, and optical properties. The material’s excellent sheet conductivity and improved valence band alignment resolve permanent issues in the development of p-type transparent conductive oxides. The applications of CuCrO_2_ include transparent optoelectronics, light-emitting diodes, thin-film transistors, and solar energy systems. Sputtered CuCrO_2_ films balance transparency and conductivity, facilitating the development of novel and sustainable devices. This review offers essential insights for guiding future research and practical implementation, positioning CuCrO_2_ as a fundamental component for next-generation transparent electronics.

## Graphical abstract

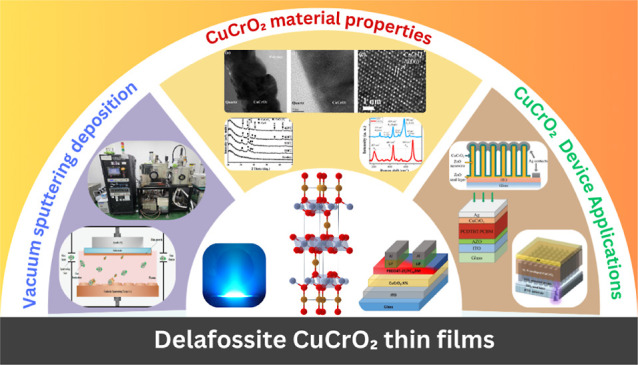

## 1 Introduction

### 1.1 Background about p-Type TCO’s

Wide-bandgap inorganic solids are commonly classified as electrical insulators and play a critical role in industrial applications such as insulators, dielectrics, and optical materials. Their wide bandgaps are attributed to the strong ionic character of the bonds between metallic cations and oxide anions, which hinders the formation of shallow donors or acceptors and promotes the localization of charge carriers.^1^ As a result, these materials have traditionally seen limited interest as conductors. Nevertheless, certain wide-bandgap oxides defy this trend and exhibit remarkable conductivity.^2^ Specifically, oxides containing p-block heavy-metal cations with ns^0^ electronic configurations (where n is the principal quantum number), such as ZnO, CdO, Ga_2_O_3_, In_2_O_3_, Ti_2_O_3_, SnO_2_, PbO_2_, Sb_2_O_5_,^3^ and their mixed oxides, can be transformed into n-type conductive oxides through electron doping.^4^ Among these, indium tin oxide (ITO), represented as In_2–*x*_Sn_*x*_O_3_, demonstrates a conductivity of up to 0.9 × 10^4^ S cm^–1^, comparable to that of conventional metals.^5^ Additionally, ITO thin films maintain optical transparency with an electron concentration of 2 × 10^21^ cm^–3^, making them highly suitable for use as transparent electrodes in flat-panel displays, solar cells, and other optoelectronic devices.^6^

The coexistence of high electrical conductivity and optical transparency is a rare phenomenon in materials, but transparent conducting oxides achieve this unique combination. TCOs, a class of degenerately doped wide-bandgap oxide semiconductors, exhibit metallic-like conductivity (∼10^4^ S cm^–1^) and insulating-like transparency (>80% in the visible spectrum).^7^ Owing to this rare combination of electrical and optical properties, TCOs play a vital role in modern optoelectronic devices such as touchscreens, displays, OLEDs, and solar cells. Recently, their functionality has expanded toward active components in transparent thin-film transistors, UV emitters, photodetectors, and gas-sensing devices^8^

Despite their versatility, the potential of TCOs is hindered by the lack of high-performance p-type TCO counterparts. The integration of p- and n-type TCOs into p–n heterojunctions could significantly enhance the functionality of transparent electronics and optoelectronics. Moreover, the increasing demand for advanced photovoltaics highlights the need for efficient p-type TCOs to improve the hole collection. The challenge in achieving p-type TCOs arises from the intrinsic electronic structure of the metal oxides. In most oxides, the valence band is dominated by strongly localized O 2p-derived orbitals. The small size and high electronegativity of oxygen makes it difficult to introduce shallow acceptors, resulting in large hole effective masses. To address this limitation, Hosono and his colleagues proposed the concept of “chemical modulation of the valence band,”^9^ using the hybridization of O 2p orbitals with closed-shell Cu 3d 10 orbitals. This approach led to the discovery of a new class of p-type TCOs based on Cu^+^ oxides, known as delafossites, represented as CuMO_2_ (M = Al, Cr, In, Sc, Y, and Ga).

### 1.2 Overview of Delafossites

Copper-based delafossites, particularly CuCrO_2_, have gained significant attention as promising p-type TCOs due to their exceptional structural, optical, and electronic properties. These materials feature a layered crystal structure that supports both electronic conductivity and optical transparency, complemented by high thermal stability and tunable visible-range optical absorption. Since the pioneering identification of their potential in 1997 ^10^, p-type delafossite TCOs have been recognized for their high hole mobility, attributed to the overlap of Cu 3d and O 2p states in the valence band.^11^ Despite these advantages, p-type TCOs face intrinsic challenges compared to their extensively studied n-type counterparts, particularly in achieving high mobility through optimized valence band alignment.^12^ Sputtered CuCrO_2_ thin films have emerged as a promising solution, offering a vacuum-based deposition technique that enables precise control over parameters, such as gas composition and annealing conditions. In particular, sputtered CuCrO_2_ thin films benefit from the strong hybridization between Cu 3d and O 2p orbitals, which results in a highly dispersive valence band. This enhanced band dispersion reduces the effective mass of holes, thereby facilitating higher carrier mobility compared with conventional p-type oxides such as NiO or CoO. Furthermore, sputtering enables precise control of oxygen stoichiometry and crystalline quality, both of which are critical for tuning valence band alignment and achieving efficient hole transport. These characteristics make CuCrO_2_ a promising candidate for next-generation optoelectronic and photovoltaic devices, where transparent p-type contacts with high conductivity are essential. The ability to deposit CuCrO_2_ films via sputtering also ensures compatibility with large-area processing and scalable device fabrication. When combined with its intrinsic wide bandgap, chemical stability, and earth-abundant elemental composition, sputtered CuCrO_2_ emerges as an attractive alternative to conventional p-type TCOs. Consequently, systematic optimization of deposition parameters and postannealing treatments can further enhance film crystallinity, carrier concentration, and mobility, paving the way toward efficient integration of CuCrO_2_ in transparent electronics, thin-film solar cells, and heterojunction-based device architectures. This method produces uniform, dense films, making it highly suitable for scalable manufacturing and integration into next-generation optoelectronic devices. Recent research has focused on advancing low-temperature deposition methods and innovative annealing processes to fabricate high-quality p-type TCOs for flexible and transparent electronics.

Beyond optoelectronics, copper-based delafossites also find applications in photocatalysis, thermoelectrics, and supercapacitors, underscoring their multifaceted potential. CuCrO_2_ is cost-effective and nontoxic and demonstrates a unique combination of transparency and electrical conductivity.^13^ As an intrinsic semiconductor with a wide bandgap exceeding 3.0 eV, it efficiently absorbs ultraviolet photons while the photon energy in the visible range is insufficient to promote electrons from the valence band to the conduction band.^14^ These properties make CuCrO_2_ an invaluable material for optoelectronic applications and a cornerstone in the development of Cu-based p-type delafossite devices. Structurally, CuCrO_2_ comprises copper in the +1-oxidation state (Cu^+^), chromium in the +3-oxidation state (Cr^3+^), and oxide ions (O^2–^), forming a composition close to 1:1:2 ratio. Its nonstoichiometric nature, often with excess oxygen, underpins its p-type conductivity.^12^

This review focuses on sputtered CuCrO_2_ thin films, highlighting their deposition techniques, postdeposition treatments, and resultant structural, morphological, electrical, and optical properties. Importance is placed on their potential applications in optoelectronic devices, sensors, and energy-harvesting technologies. Although several review articles have summarized the development of p-type TCOs, they typically adopt a broad perspective—either covering the entire family of delafossites (e.g., CuMO_2_, where M = Cr, Al, Ga, etc.) or discussing CuCrO_2_ prepared by various deposition techniques such as pulsed laser deposition (PLD), chemical vapor deposition, or sol–gel routes. While these surveys provide valuable overview of material properties and general progress in the field, they often lack a focused analysis on sputtered CuCrO_2_ films. In particular, the critical influence of sputtering parameters on crystallinity, defect formation, carrier mobility, and subsequent device integration remains insufficiently addressed. The present review aims to bridge this gap by concentrating exclusively on sputtering-based strategies for CuCrO_2_ thin films. By systematically evaluating how process conditions govern the structural, optical, and electronic characteristics of sputtered CuCrO_2_, this work provides a targeted perspective that complements existing reviews and offers practical insights for advancing sputtered p-type TCOs in transparent electronics and photovoltaic applications. By providing a comprehensive analysis of these aspects, this Review aims to guide future research and foster advancements in the development of high-performance CuCrO_2_-based TCOs for next-generation applications.

### 1.3 Delafossite Crystal Structure

The delafossite structure, represented by the general formula ABO_2_, exhibits distinctive structural and electronic properties that render it highly suitable for applications in optoelectronics and TCOs. In this structure, the A-site is typically occupied by a monovalent metal, such as copper (Cu) or silver (Ag), while the B-site hosts a trivalent metal, including aluminum (Al), chromium (Cr), iron (Fe), or gallium (Ga). This combination of A- and B-site elements contribute significantly to the material’s electronic behavior and optical properties.

The delafossite structure is characterized by two primary polymorphs, which is shown in Figure 1, each distinguished by unique stacking sequences and crystallographic symmetries. These structural variations have a direct impact on the material’s electronic and optical properties:

•Rhombohedral Polymorph (Space Group *R*3̅*m*): In the rhombohedral polymorph, the BO_6_ octahedral layers are stacked in an ABC sequence. The A-site atoms are linearly coordinated between these octahedral layers. This linear coordination facilitates strong electronic interactions between the A- and B-site atoms, contributing to enhanced p-type conductivity. The hybridization between the A-site 3d orbitals (for Cu) or 4d orbitals (for Ag) and the B-site orbitals play a crucial role in modulating the electronic band structure.^15^
•Hexagonal Polymorph (Space Group *P*6_3_/*mmc*): The hexagonal polymorph features an ABAB stacking sequence of BO_6_ octahedral layers. In this configuration, the neighboring octahedral layers are oppositely oriented, which alters the orbital alignment and, consequently, the electronic properties. This polymorph exhibits different conduction pathways compared to the rhombohedral structure, impacting its conductivity and optical behavior.^16^

**Figure 1:**
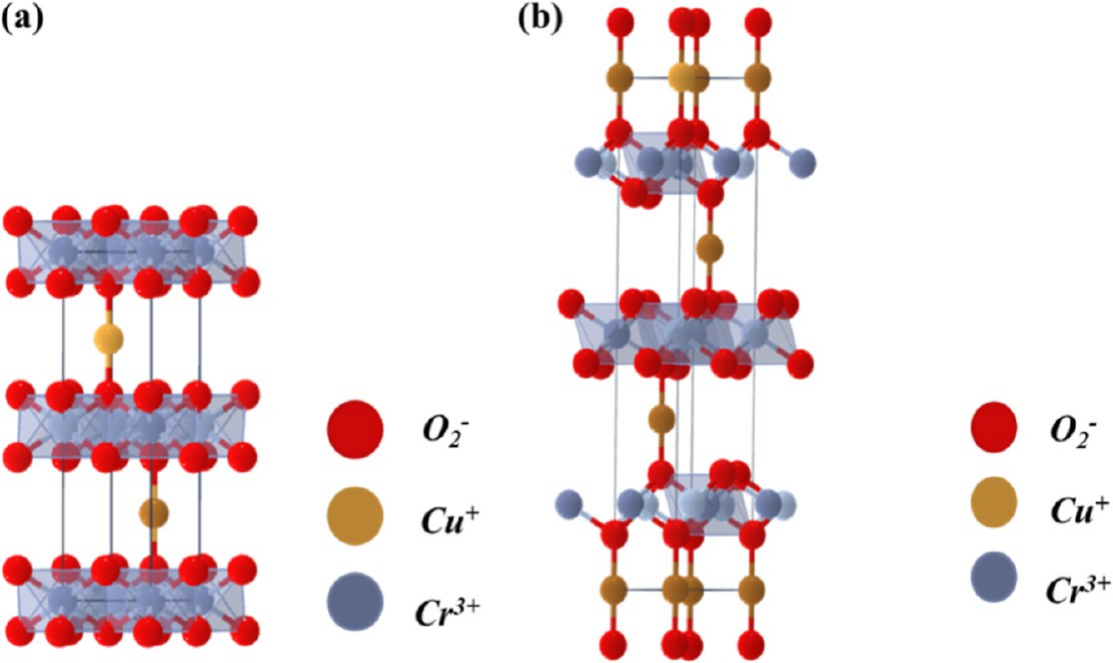
Schematic representation of delafossites: (a) hexagonal polymorph and (b) rhombohedral polymorph from materials project.

The energy difference between these two polymorphs is relatively small, making the synthesis conditions a determining factor for obtaining one structure over the other. Variables such as temperature, pressure, and chemical composition during the synthesis can drive the formation of either polymorph. This structural flexibility allows delafossites to be tailored for specific electronic and optical properties, including bandgap tuning and conductivity optimization.

The unique layered arrangement of delafossite structures, combined with their coordination chemistry, further enhances their versatility. For example, in CuCrO_2_, the interplay of Cu^+^ ions at the A-site and Cr^3+^ ions at the B-site which can be observed visually in Figure 2, along with the oxygen sublattice, supports a wide bandgap and p-type conductivity. Additionally, the ability to modulate synthesis conditions to control the structural polymorph opens avenues for fine-tuning their performance in targeted advanced optoelectronic applications.

**Figure 2:**
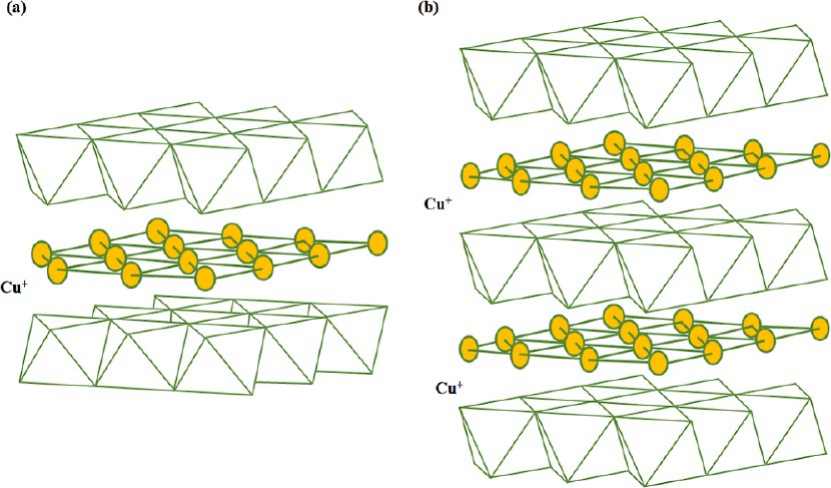
Crystal structure illustrations of CuCrO_2_ polymorphs: (a) 2H–CuCrO_2_ and (b) 3R–CuCrO_2_.

The electronic properties of CuCrO_2_, a delafossite compound, are closely linked to its crystal structure, which can exist in two polymorphs. The rhombohedral *R*3̅*m* structure features [CrO_6_] octahedral layers stacked in an ABCABC sequence with Cu atoms linearly coordinated by O atoms between layers, facilitating efficient charge transfer. The hexagonal *P*6_3_/*mmc* polymorph also has [CrO_6_] layers but stacked in an ABAB sequence as shown in Figure 3, where neighboring layers are oppositely oriented. Both structures exhibit similar energies, so small synthesis changes can yield either form. This structural flexibility allows CuCrO_2_ to be tuned for desired properties, making it suitable for thin-film applications, where minor adjustments enhance electrical and optical performance.

**Figure 3:**
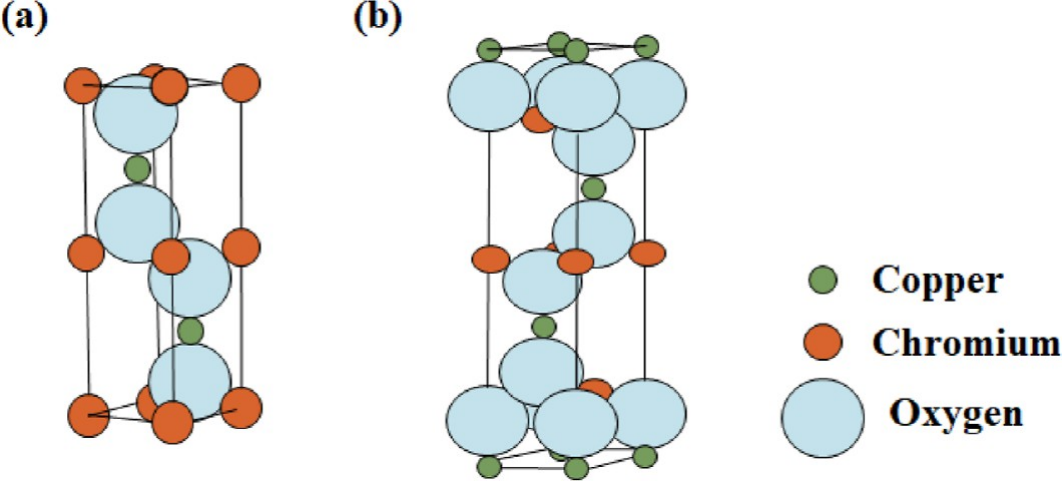
Schematic of CuCrO_2_ polymorph unit cells. (a) Hexagonal −2*H* polymorph (*P*6_3_/*mmc*) and (b) Rhombohedral −3R polymorph (*R*3̅*m*).

### 1.4 Deposition of Delafossite CuCrO_2_ Thin Films

A variety of approaches have been employed to deposit CuCrO_2_ thin films, generally classified into chemical-^17^ and vacuum-based processes. Chemical deposition methods, including spray pyrolysis,^18^ dip coating,^19^ sol–gel processing,^20^ and chemical vapor deposition,^21^ offer economical solutions for large-area thin films but frequently necessitate supplementary postdeposition treatments to enhance film characteristics.

Vacuum deposition methods, such as RF sputtering, PLD,^22^ and atomic layer deposition (ALD),^23^ are esteemed for their accuracy and consistency. PLD is recognized for generating high-quality films with superior stoichiometric precision, whereas ALD specializes in the fabrication of ultrathin, conformal coatings suitable for particular applications. Sputtering, a prevalent vacuum-based technique, has been utilized in multiple variants, including DC, RF, hybrid, and reactive magnetron sputtering, for the deposition of CuCrO_2_ thin films as shown in Figure 5.

These deposition procedures present unique advantages and trade-offs, contingent upon the required film properties, scalability, and application specifications, rendering them essential for the evolution of CuCrO_2_-based technologies. Figure 4 shows the classification of (a) number of papers published in CuCrO_2_ using various deposition methods, (b) number of CuCrO_2_ papers publications using vacuum-based Sputtering Deposition—both these data were extracted from the SCOPUS Web site excluding other publication platforms.

**Figure 4:**
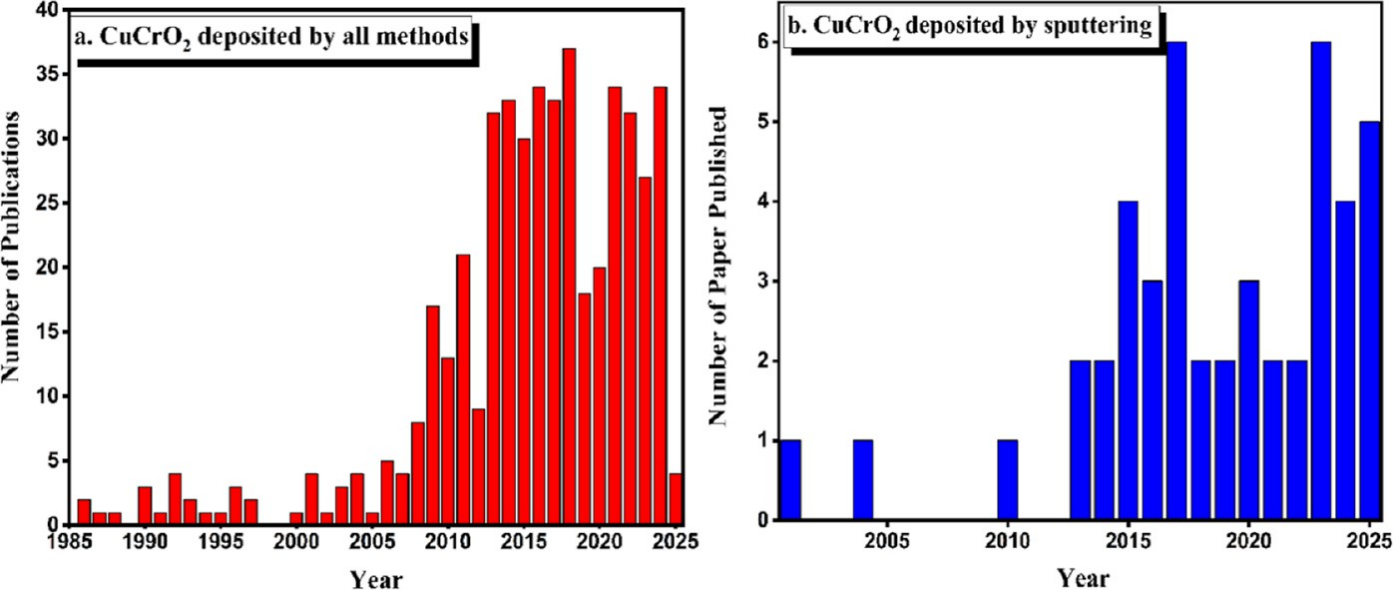
(a) Number of papers published in CuCrO_2_ using various deposition methods and (b) number of CuCrO_2_ papers using vacuum-based sputtering deposition (Data for both extracted from SCOPUS until 2025).

**Figure 5:**
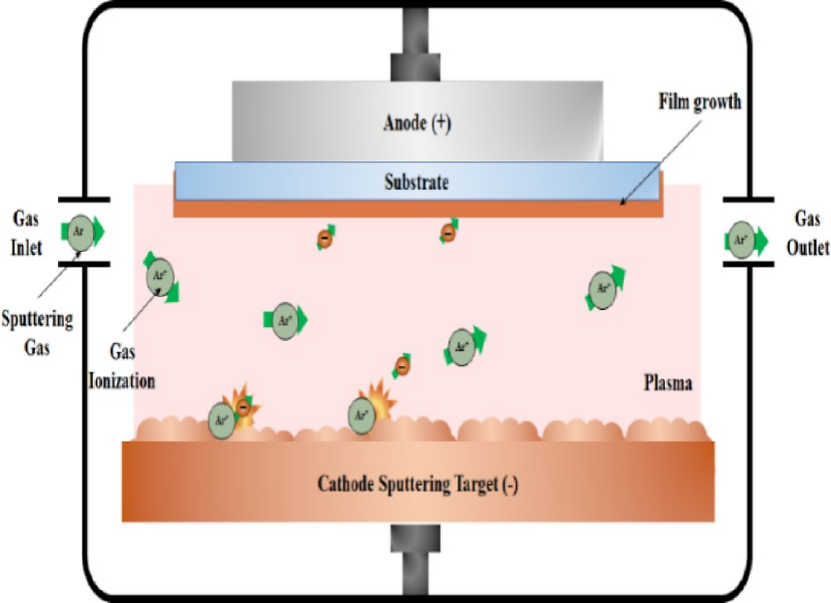
Working schematic of sputtering technique.

### 1.5 Sputter-Deposited Delafossite CuCrO_2_ Thin Films

This review focuses on the sputtering deposition method for fabricating CuCrO_2_ thin films, emphasizing its effectiveness in producing high-quality TCOs. Among various deposition techniques, sputtering stands out for its ability to achieve precise control over film thickness, composition, and uniformity across large areas. This makes it a versatile method compatible with various substrate materials and suitable for a wide range of applications. Sputtering has been a common technique for material fabrication, particularly since its adoption in optical disc applications more than two decades ago. Despite significant advancements in sputtering technology and its widespread understanding, it remains essential to revisit some of its fundamental principles.^24^

The sputtering process as shown in Figure 5 involves the ejection of atoms from the target material caused by plasma bombardment. To generate this plasma, the sputtering chamber is first evacuated using a vacuum pump. Argon (Ar) is commonly used as a working gas due to its inert nature and cost-effectiveness. The process operates under vacuum conditions, allowing precise control over deposition parameters, such as target power, substrate temperature, working pressure, and gas composition (e.g., oxygen and argon). These variables critically influence the structural, morphological, optical, and electrical properties of the resultant CuCrO_2_ films, enabling their optimization for specific applications.^25^

Three key experimental parameters govern the sputtering process:

- Base pressure—The vacuum pressure before sputtering begins.
- Working pressure—The pressure established after maintaining a constant flow of Ar gas.
- Applied power—Used to generate the plasma, which can be DC for conductive targets (e.g., metals) or RF for a broader range of materials, including insulators.

A notable advantage of sputtering is its scalability, making it ideal for industrial fabrication of high-quality TCOs.^26^ Its ability to produce films with exceptional homogeneity across large areas further cements its role as a critical technique for the large-scale production of transparent electronic devices. Additionally, postdeposition annealing is frequently employed to enhance the crystallinity of sputtered films, improving their optoelectronic properties, such as electrical conductivity and optical transparency. While sputtering offers clear advantages such as precise control of deposition parameters, uniform and conformal film growth, and compatibility with large-area and industrial-scale processing, it is equally important to acknowledge its limitations when it is applied to CuCrO_2_ thin films. One significant challenge lies in the composition control of the Cu/Cr ratio, as even minor deviations from the ideal stoichiometry can strongly influence the structural and electronic properties of the resulting films. For example, an excess of copper can lead to the formation of secondary phases, such as CuO or Cu_2_O, while an excess of chromium can promote the growth of insulating Cr_2_O_3_ domains. Both scenarios adversely affect the conductivity and transparency of the films and may also introduce undesired band alignment at heterojunction interfaces. Additionally, reactive sputtering processes, which are often employed to incorporate oxygen, can suffer from target poisoning. This occurs when oxygen reacts with the metallic target surface, forming an oxide layer that alters the sputtering yield, reduces deposition rates, and leads to unstable plasma conditions.

Another inherent limitation of sputtering is the relatively low deposition rate compared to techniques such as chemical vapor deposition or spray pyrolysis, which may slow down large-scale manufacturing. Moreover, nonoptimized sputtering conditions—such as improper substrate temperature, gas pressure, or oxygen partial pressure—can result in the generation of point defects, including oxygen vacancies, interstitials, and antisite defects. These imperfections can act like carrier traps, scattering centers, or recombination sites, thereby degrading hole mobility and overall film performance. Furthermore, the high-energy bombardment of particles during sputtering may introduce lattice strain or even amorphous regions, particularly in films deposited at lower substrate temperatures. Such microstructural imperfections not only reduce crystallinity but also complicate postdeposition annealing strategies, as defect healing and phase stabilization often require fine-tuned thermal treatments.

Taken together, these factors highlight that while sputtering is a powerful and versatile method for fabricating CuCrO_2_ thin films, achieving optimal film quality necessitates meticulous control over target composition, plasma chemistry, and growth conditions. Understanding and mitigating these limitations are therefore essential for harnessing the full potential of sputtered CuCrO_2_ in transparent electronics and optoelectronic devices.

Another significant feature of sputtering is the possibility of cosputtering,^27^ which involves the simultaneous use of multiple targets. This enables the deposition of alloy films composed of two or more elements. These alloys can be formed either through a single alloy target or by cosputtering individual elemental targets. This flexibility is particularly advantageous for tailoring the composition of CuCrO_2_ films to achieve specific properties, such as p-type conductivity. Sputtering plays a crucial role in depositing nonstoichiometric CuCrO_2_ films, which are vital for attaining p-type conductivity. By meticulously regulating oxygen content or employing deliberate doping strategies, sputtering enables precise refinement of the electronic properties, meeting the demands of next-generation transparent electronic devices.

Numerous research studies in the literature have investigated the effect of doping on the enhancement of CuCrO_2_ thin films, specifically how various dopants affect the material’s electrical and optical properties.^28^ Doping introduces supplementary charge carriers, such as electrons or holes, which markedly enhance the conductivity of CuCrO_2_ films, rendering them more appropriate for applications necessitating p-type Transparent Conductive Oxides. Nitrogen doping has also been explored to enhance the hole concentration by replacing oxygen atoms in the lattice. This substitution boosts electrical conductivity and enables the adjustment of bandgap features. Metallic dopants including Mg^29^ and Zn,^30^ when integrated into the CuCrO_2_, have demonstrated enhancements in hole mobility and concentration. This enhancement is probably attributable to the alignment of their atomic orbitals with those of copper, facilitating efficient charge carrier transfer. These dopants can alter the Fermi level, improving p-type conductivity while minimally impacting optical transparency.

Alternative dopants, including Fe^31^ and Ni,^32^ have been documented to alter the defect architecture of CuCrO_2_, promoting the emergence of acceptor states that enhance the density of charge carriers. Moreover, regulated doping can diminish vacancy flaws in CuCrO_2_ films, thereby enhancing their thermal and chemical durability along with their adherence to diverse substrates. Optimizing dopant concentration is essential since excessive doping may result in adverse consequences such as phase separation, structural distortion, or diminished optical transmittance due to free carrier absorption. Meticulous adjustment of doping parameters guarantees that CuCrO_2_ films satisfy the stringent conductivity and transparency standards for optoelectronic applications including transparent electrodes, photovoltaic cells, and light-emitting devices. The sputtering parameters and doping conditions for the deposition of CuCrO_2_ thin films are listed in Table 1, offering a detailed overview of the approaches used to attain the desired film properties. This systematic method emphasizes the crucial function of doping in optimizing the performance of CuCrO_2_ for advanced electrical and optoelectronic technologies.

**Table 1:** Sputtering Parameters of CuCrO_2_ Thin Films Listed Year Wise from 2013 to 2025

Type of sputtering	Target used/target type	Substrate material	Substrate temp	Working/Sputtering pressure (base)	Sputtering Power	Substrate to Target distance	Sputtering Time (minutes)	Sputtering gas and pressure	Annealing Temperature	Year	ref
RF sputtering	ceramic CuCrO_2_ target	alumina tubes	600 °C	2.0 × 10^–2^ mbar	70 W	5 cm		Ar gas 50 sccm		2025	^33^
RF magnetron sputtering	Cu,Cr-doped powder targets	Ti alloy and Si(100)		7.5 × 10^–7^ Torr (Base)	140 W		60	Ar/75 sccm		2025	^34^
RF magnetron sputtering	Cu Cr metal target	Si(100)	room temperature	1 × 10^–7^Torr (Base)	69 W		45	Ar/39 sccm		2025	^35^
RF&DC Magnetron Sputtering	Cu/Cr metal targets	quartz substrate	300 °–400 °C	10 mTorr	For Cu: 10–40 W and Cr/100 W			Ar and O_2_ = 20:1 sccm	800 °C	2024	^36^
RF magnetron sputtering	polycrystalline target Mg/CuCrO_2_	glass substrates	300 ̊C		150 W			N_2_/(N_2_+Ar) = 40%	250 °C	2024	^37^
DC magnetron sputtering	CuCr with at. ratio of 1:1	fused silica substrates	400 °C	5 × 10^–3^ Torr	200 W	9 cm	15	Ar gas	500–800 °C	2023	^38^
DC magnetron sputtering	Mg/CuCrO_2_	soda-lime glass	450 °C	3 × 10^–3^ Torr	120 W	6 cm	10	Ar gas; 3 × 10^–3^ Torr		2023	^39^
RF magnetron sputtering	Cu_2_O and Cr_2_O_3_ targets	Quartz	400 °C	5 × 10^–7^ Torr(Base)	50 W	5 cm		Ar	600–900 °C	2023	^40^
RF Magnetron sputtering	Cu_2_O and Cr_2_O_3_ targets	quartz	400 °C	5 × 10^–7^ Torr(Base)	50 W			Ar gas	650 °C	2023	^41^
DC sputtering	Mg-doped CuCrO_2_	glass	300 °C	3 × 10^–3^ Torr	120 W	6 cm	40–80		500 °C	2022	^42^
Pulsed DC and RF magnetron sputtering	copper–chromium alloy target	fused quartz						Ar and N_2_ gases		2021	^43^
RF magnetron sputtering	ceramic CuCrO_2_ target	C-face sapphire	500 °C	10^–6^ Pa (Base)	150 W	110 mm		N_2_ and Ar; 1.0 Pa		2021	^44^
RF sputtering	Mg-Doped CuCrO_2_ target	quartz		1.2 × 10^–1^ mBar	150 W	5 cm	15	Ar gas	900 °C	2021	^45^
RF magnetron sputtering	ceramic CuCrO_2_	C-face sapphire	500–800 °C		150 W	110 mm or 11 cm		H_2_/N_2_ gas; 1000 Pa		2020	^46^
DC Sputtering	Cu,Mg and Cr metallic targets	P type Si(100)		0.9 Pa	29, 27, 250 W			Ar and O_2_ 90:10 sccm	1023 K at vacuum for 30 min	2020	^47^
RF and DC sputtering	Cu and Cr targets	fused silica		3.0 × 10^–3^ Torr	10–52 W	50 mm		Ar and O_2_ gases	Ar atmosphere; 700 °C	2019	^48^
DC and RF magnetron sputtering	CuCrO_2_	quartz	700 °C	2 × 10^–5^ pa		5 cm	60	Ar and N_2_ gas; 2 × 10^–5^–5 × 10^–2^ Torr	625 °C; 2 h	2018	^49^
Reactive RF magnetron sputtering	ceramic CuCrO_2_ target	c-face sapphire			150 W	110 mm or 11 cm		Ar/N_2_ gas; 1.0 Pa		2018	^50^
RF-sputtering	Mg-doped CuCrO_2_ Target	fused silica			50 W	5 cm		Ar; 0.5 Pa	thermal annealing; N_2_ Atmosphere; 550 °C	2018	^51^
RF Magnetron Sputtering	ceramic CuCrO_2_ Target	fused quartz	450 °C		150 W	5 cm		Ar and N_2_gas; 1 Pa	air Atm.; 450 and 550 °C	2017	^52^
DC reactive magnetron sputtering	Pure Cu, Cr, and Mg target	alumina, quartz	1023–1123 K	0.95 Pa				Ar and O_2_ gases		2017	^53^
RF magnetron sputtering	CuCrO_2_ ceramic target	c-face sapphire			150 W			Ar and N_2_; 1.0 Pa		2017	^54^
DC magnetron cosputtering	Cu and Cr target	fused silica				50 mm	40	Ar and O	Ar atmosphere; 800 °C; 30–240 min	2016	^55^
Pulsed DC sputtering	copper–chromium	quartz		4 × 10^–3^ Torr	250 W		25	O_2_	700 °C; 10 min	2016	^56^
RF-sputtering	CuCrO_2_ target	quartz and silicon	450–800 °C		0.9 W/cm^2^	5 cm		Ar gas; 0.5 Pa	450 and 600 °C	2015	^57^
DC magnetron sputtering	copper–chromium alloy target	sapphire	400 °C	0.5 Pa	100 W	∼5 cm		Ar gas	900 °C	2015	^58^
Pulsed DC and reactive magnetron sputtering	copper–chromium target	quartz glass	25 °C	2.0 × 10^–6^ Torr(Base)		100 mm	50	Ar and O_2_ gases; 3.0 × 10^–3^ Torr	Ar atmosphere; 600 °C	2014	^59^
DC Magnetron Sputtering	Mg-Doped CuCrO_2_ Target	P type Si(100)		4 × 10^–4^ Pa and 0.5 Pa	100 W	60 mm		Ar/O_2_ gas at 3:1 ratio	N_2_ environment at 700 °C	2014	^60^
Reactive magnetron sputtering	copper–chromium alloy	quartz glass		4.0 × 10^–1^ Pa to8.0 × 10^–4^ Pa	200 W		50	Ar gas; 550 to 625 °C	Ar Atmosphere; 600 °C	2013	^61^
RF magnetron sputtering	CuCrO_2_ target	corning eagle/black glass	200 °C	2.93 to 3.33 × 10^–2^ mbar	150 W			Ar/O_2_; 30 sccm/8 sccm (gas flow rate)	400 °C	2013	^62^

The subsequent section explores the extensive optoelectronic characteristics of sputtered CuCrO_2_ thin films, offering a thorough examination of each feature based on current research. The properties comprise structural, optical, electrical, and morphological aspects, which collectively ascertain the material’s compatibility for advanced optoelectronic applications. We outline the principal findings from relevant investigations for each property, highlighting the impact of deposition parameters—namely, substrate temperature, working pressure, gas composition, and postdeposition treatments—on the resultant film properties. This systematic method facilitates a comprehensive understanding of the interaction among various parameters to enhance the performance of CuCrO_2_ thin films.

Alongside the comprehensive research, we present a consolidated summary in specific sections that outline the ideal circumstances necessary for attaining the delafossite phase—a pivotal element for assuring superior optical transparency and p-type conductivity. This includes a comprehensive examination of characterization methods, including X-ray diffraction and Raman spectroscopy for structural phase confirmation; UV–vis spectroscopy for assessing optical transparency and bandgap; Hall effect measurements for evaluating electrical conductivity and carrier type; XPS for evaluating the compositional properties; and Atomic Force Microscopy (AFM) and scanning electron microscopy for surface morphology analysis. By linking these features with their corresponding fabrication conditions, we sought to build a definitive structure for optimizing sputtering procedures. This data synthesis highlights the essential function of parameter control in producing high-quality CuCrO_2_ thin films and serves as a significant resource for researchers aiming to reproduce or improve these findings in further investigations.

## 2 Properties of Delafossite CuCrO_2_ Thin Films

### 2.1 Structural Analysis

#### 2.1.1 X-ray Diffraction

X-ray diffraction serves as a fundamental analytical technique widely employed for the examination of phase identification, crystallographic structure, and other structural properties of materials. It operates on the principle of Bragg’s law, which establishes a relationship between the diffraction angle, the lattice spacing within a crystal, and the wavelength of X-rays.^63^ XRD plays a pivotal role in deciphering the stability of CrO and the influence of Cu doping on the crystalline structure of CuCrO_2_. In the context of CuCrO_2_ thin films, two dominant XRD peaks are typically observed around 31° and 36°, corresponding to the (006) and (012) planes, respectively. The annealing process is significant in this regard. High-temperature annealing can enhance the structural quality of the material, decrease dislocation densities, and further alleviate residual strain. The term “dislocation densities” refers to the cumulative length of dislocations present per unit volume of a material.

Sundaresh et al. studied the XRD patterns of RF-sputtered CuCrO_2_ thin films annealed at different temperatures. The nanocrystalline CuCrO_2_ thin films generally form at higher annealing temperatures (above 600 °C), which is evident from the amorphous nature of films annealed at 500 °C as shown in Figure 6a. Prominent diffraction peaks appeared only at annealing temperatures above 600 °C and at annealing temperature of 600 °C; characteristic peaks of CuO (indicated as “o” in Figure 6b), Cr (indicated as “#” in Figure 6), and CuCr_2_O_4_ (indicated as “+” in Figure 6) were identified. However, the CuO and CuCr_2_O_4_ phases in films are not suitable for TCO applications. With an increase in the annealing temperature to 650 °C, all the peaks disappeared, leading to the promotion of the pure-phase delafossite nature of CuCrO_2_ thin films (indicated as “*” in Figure 6). Further increases in the annealing temperatures led to the disintegration of single-phase CuCrO_2_. Additional Cr peaks started to appear in the thin films, annealed at 750 °C along with CuCrO_2_ peaks. Since Cr is characteristically a high-temperature species, the single-phase CuCrO_2_ disintegrates, and the Cr peak is observed at 750 °C, which appears as Cr_2_O_3_ with a further increase in the annealing temperature. At annealing temperatures of 800 and 900 °C, Cr_2_O_3_ peaks (indicated as “•” in Figure 6) were identified in addition to CuCrO_2_ peaks. It is safe to conclude that both CuO and CuCr_2_O_4_ are comparatively lower temperature phases.^40^

**Figure 6:**
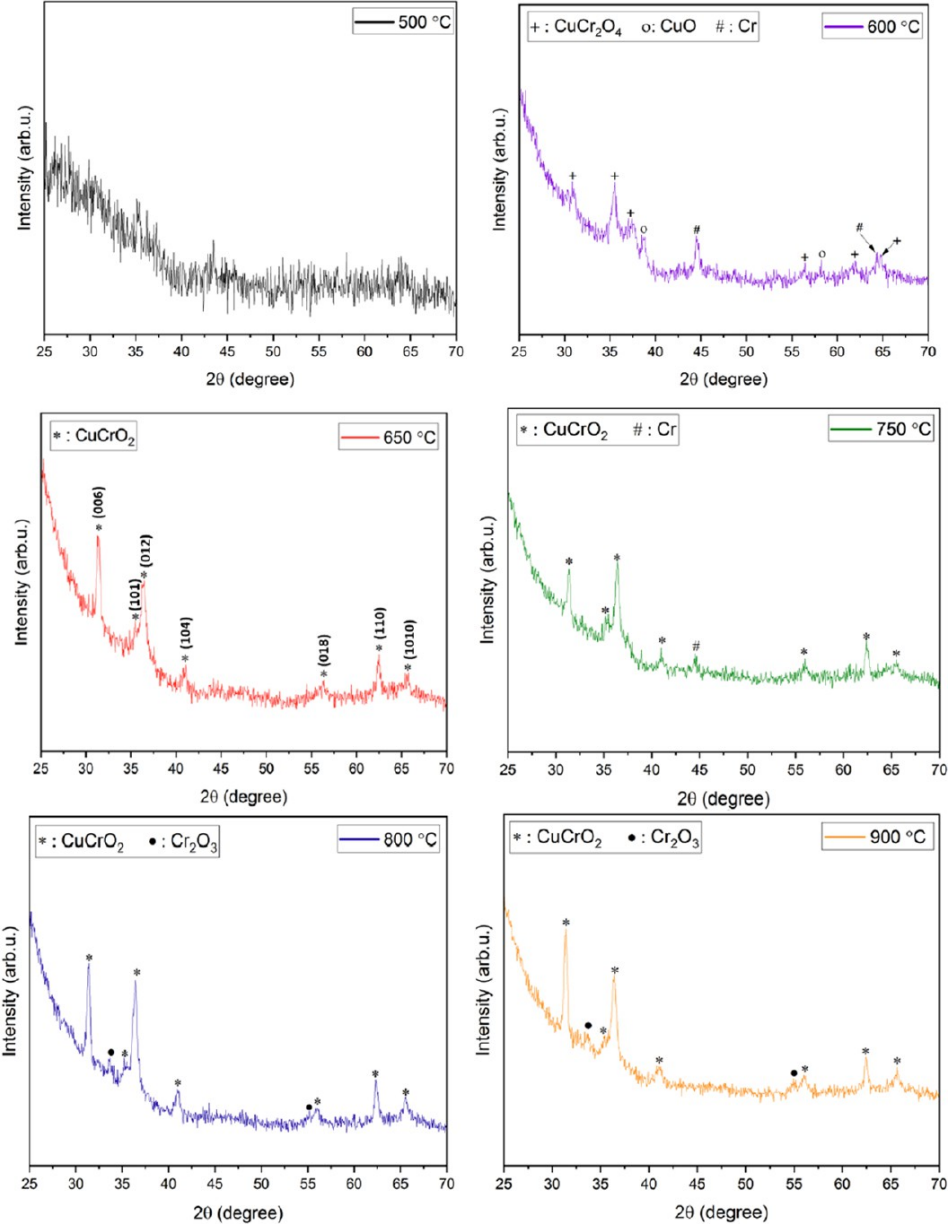
XRD patterns of CuCrO_2_ films annealed at (a) 500 °C, (b) 600 °C, (c) 650 °C, (d) 750 °C, (e) 800 °C, and (f) 900 °C. Reprinted from ref 40 under the terms of the Creative Commons (CC BY 4.0) license.

Yu et al.^61^ also revealed that the Pulsed DC-sputtered CuCrO_2_ films exhibited diffraction peaks corresponding to the (006) and (012) planes, as shown in Figure 7. The full width at half-maximum (fwhm) of the (006) peak was employed to estimate the average crystallite size using the Debye–Scherrer equation. The calculated average grain sizes for films annealed at 600 and 625 °C were 20.73 and 29.34 nm, respectively. This increase in the crystallite size at higher annealing temperatures indicates a reduction in grain boundary density, which is beneficial for carrier transport. From these observations, it is evident that the delafossite CuCrO_2_ phase stabilizes at higher temperatures compared to secondary phases such as CuO and CuCr_2_O_4_. The thermodynamic driving force for this phase formation can be understood in terms of the free energy of the CuCrO_2_ system: at annealing temperatures ≥600 °C, the free energy of the CuCrO_2_ phase decreases sufficiently to favor its nucleation. However, the transformation does not complete instantaneously at 600 °C since the reaction kinetics are still limited by atomic diffusion. When the annealing temperature is increased to 625 °C, enhanced atomic mobility accelerates diffusion rates, resulting in a more rapid transformation into the CuCrO_2_ phase and pronounced grain growth, even within short annealing durations.

**Figure 7:**
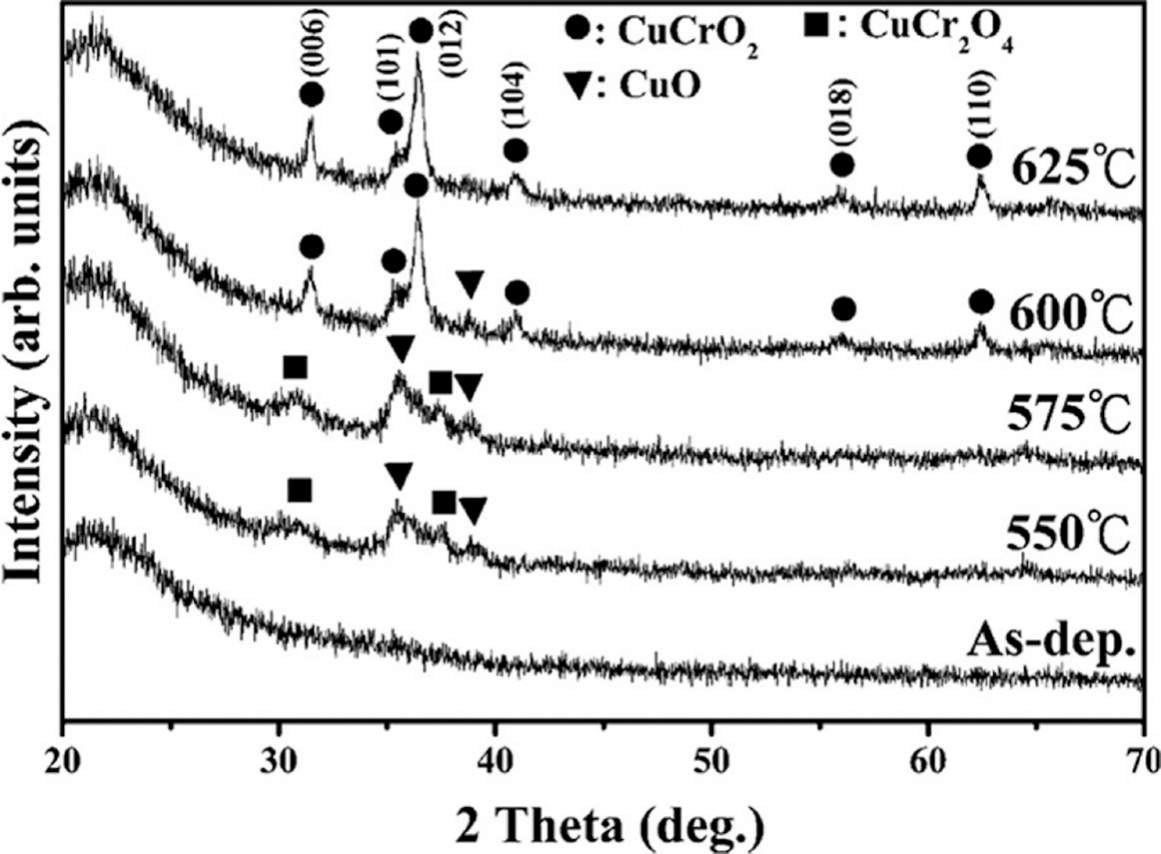
XRD patterns of the as-deposited film and films annealed at 550, 575, 600, and 625 °C. Reproduced with permission from ref 62, Copyright 2013, Elsevier.^61^

A notable distinction of CuCrO_2_, compared to other delafossite oxides, lies in its relatively low phase formation temperature. For instance, CuCrO_2_ thin films typically require postdeposition annealing at 900–1100 °C to achieve a stable delafossite structure, and CuGaO_2_ also demands similarly elevated processing conditions. In contrast, CuCrO_2_ achieves crystallization and phase stabilization at substantially lower temperatures (≈600–650 °C), representing a clear advantage for low-temperature processing. This comparatively mild thermal budget not only reduces processing costs but also enables the integration of CuCrO_2_ with temperature-sensitive substrates and multilayer device architectures. Therefore, CuCrO_2_ emerges as a particularly versatile delafossite oxide, offering both favorable transport properties and compatibility with practical thin-film device fabrication strategies.^61^

#### 2.1.2 Glancing Angle Incidence XRD (GA-XRD)

Chiu et al. compared the GA-XRD patterns of CuCrO_2_ for two different RF-sputtered samples: one as-deposited and other with postdeposition treatment (PDA). The PDA process involved two stages. The first stage uses a forming gas (5% H_2_ + 95% Ar) and is conducted at 400 °C for 15 min. The second stage uses an inert gas (N_2_) and is carried out at a sintering temperature of 600 °C for 1 h. The GA-XRD pattern of the films showed clear peaks belonging to CuCrO_2_, (006), (012), and (110), indicating a crystalline structure. From Figure 8, we can clearly observe the highest intensity peak belongs to the group of (006) confirming the presence of the delafossite structure of CuCrO_2_.^62^

**Figure 8:**
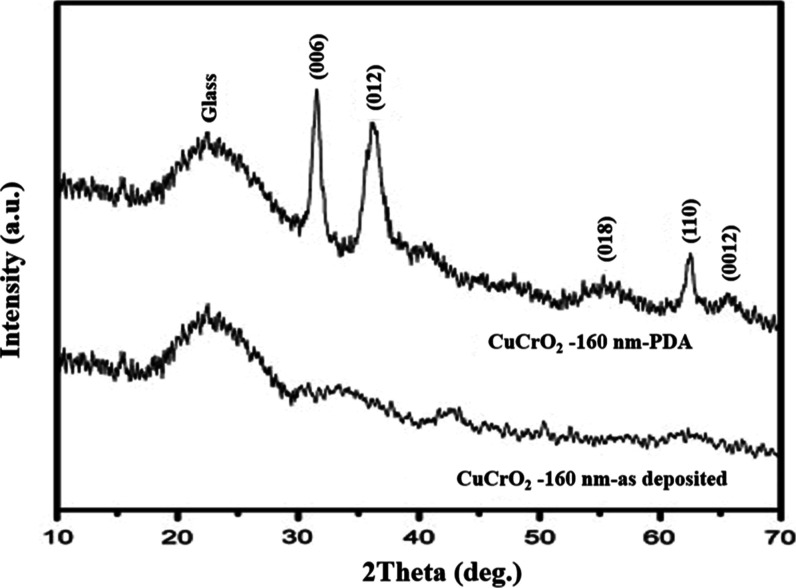
GA-XRD pattern of CuCrO_2_ films on the glass substrate. Reproduced with permission from ref 62. Copyright 2013, Elsevier.

#### 2.1.3 Out-of-Plane X-ray Diffraction

Chiba et al. studied the Reactive RF-sputtered films using Out-of-Plane XRD patterns of the as-deposited films, and the films calcined from 500 to 900 °C. They also varied the α_N_2__ (ratio of the N_2_ partial pressure) from 0 to 90% (0, 40, 70, and 90%). The Out-of-Plane XRD patterns of the as-deposited and calcined CuCrO_2_ films analyzed and their dependence on temperature coefficient (T_C_) are shown in Figure 9. Most of the characteristic peaks were observed between 37° and 45°, and 64°, which are the various diffractions from Al_2_O_3_ (006) and (009). The (006) and (009) diffraction planes correspond to the sapphire (Al_2_O_3_) substrate used in these measurements. For CuCrO_2_ films (α_N_2__ = 0%), no diffraction was observed from the film up to a T_C_ of 500 °C, and the diffraction peak from CuCrO_2_ (006) at 31.4° appeared at 550 °C. The intensity increased with T_C_ up to 700 °C and decreased slightly to 750 °C and above. For the nitrogen-doped CuCrO_2_ (CuCrO_2_:N) films deposited at αN_2_ of 40%, the dependence of the intensity on T_C_ was almost the same as that of CuCrO_2_ films. However, the (006) diffraction intensity for the CuCrO_2_:N films at an α_N_2__ of 40% became twice as high as that for the CuCrO_2_ films. On the other hand, with an α_N_2__ of 70%, a weak and broad diffraction peak appeared around 38° in the as-deposited films. The peak shifted to a lower angle and became broader at a T_C_ of 500 °C and disappeared around 600 °C. Considering these facts, candidates for this peak were metal nitrides such as CrN and CuN_3_. The diffraction peaks from CrN (111) and CuN_3_ (202) would appear at 2θ of 37.6° and 38.4°, respectively.^50^

**Figure 9:**
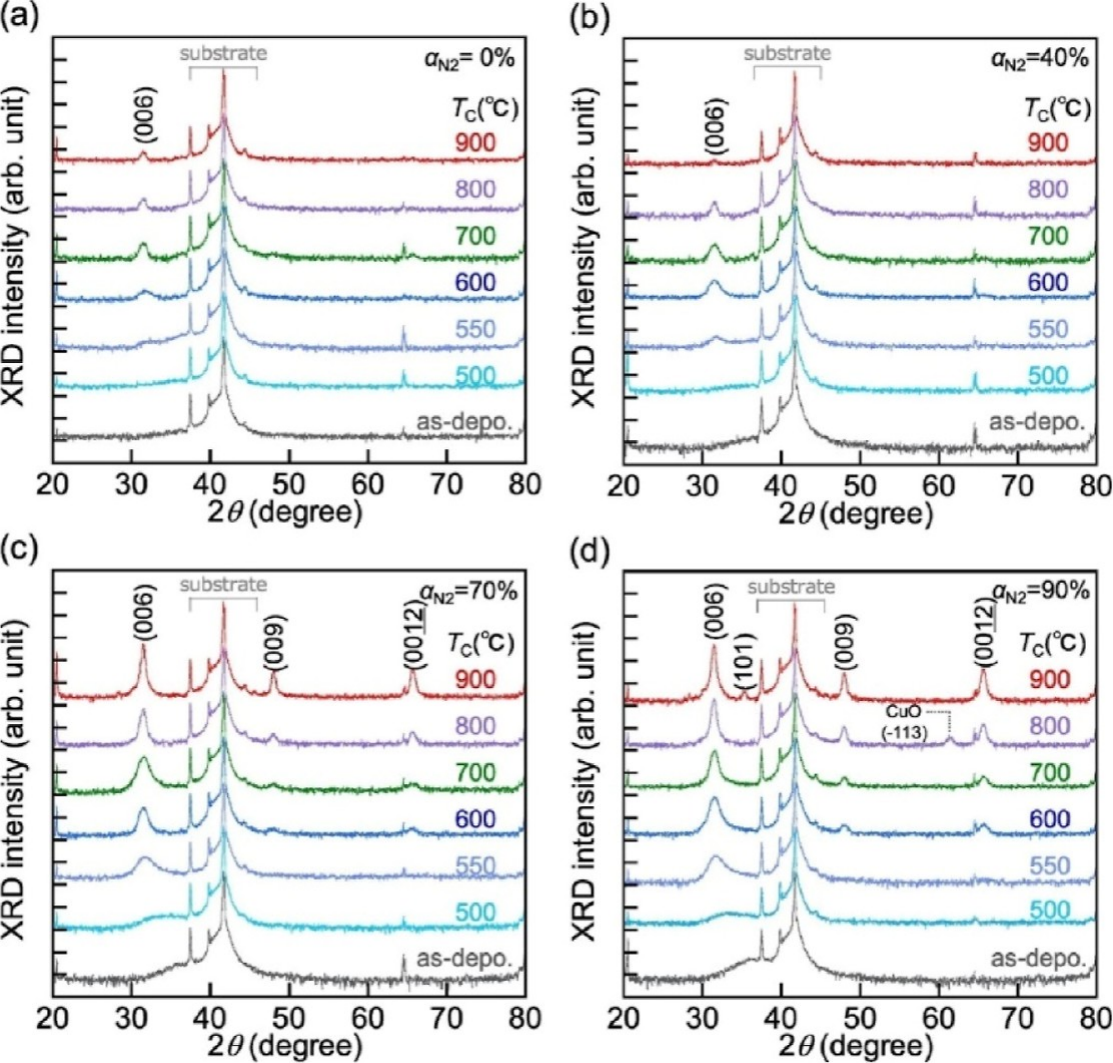
Out-of-Plane XRD patterns of both as-deposited films and calcined films (temperatures from 500 to 900 °C) for different α_N_2__ values: (a) 0%, (b) 40%, (c) 70%, and (d) 90%. Reproduced with permission from ref 50. Copyright 2018, Elsevier.

Okada et al. also conducted a study on the Out-of-Plane XRD pattern of RF-sputtered CuCrO_2_ thin films that were deposited on a c-face sapphire substrate (shown in Figure 10). Their findings were in alignment with all the previous discussions.^44^

**Figure 10:**
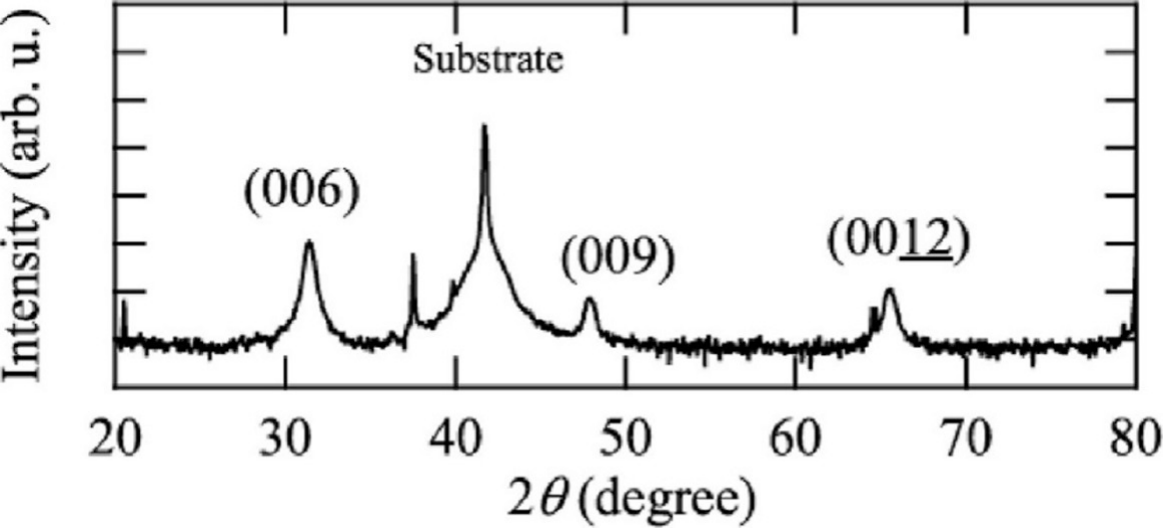
XRD patterns of CuCrO_2_ films on c-face sapphire substrates. Reproduced with permission from ref 44. Copyright 2021, Elsevier.

After analyzing various XRD studies on CuCrO_2_ thin films, it can be concluded that the primary diffraction peaks corresponding to the (006) and (012) planes, located at 2θ angles of 31° and 36.48°, respectively, are characteristic of the delafossite structure of CuCrO_2_. Most investigations have utilized RF magnetron sputtering with CuCrO_2_ compound targets, as this method facilitates the formation of the delafossite phase more efficiently and in a more controlled manner compared to cosputtering with separate Cu and Cr metallic targets. The latter often leads to the formation of secondary phases, such as CuO and CuCr_2_O_4_, and undesirable impurities on the substrate surface, which are detailed in Table 2. The formation of high-quality CuCrO_2_ thin films is typically achieved at elevated deposition temperatures exceeding 600 °C, which are notably higher than the temperatures required for CuO and CuCr_2_O_4_ formation.^61^ Additionally, the partial pressure of nitrogen (N_2_) during deposition significantly impacts the film’s crystallinity, with higher N_2_ ratios promoting the incorporation of nitrogen into the lattice, leading to CuCrO_2_:N formation.^50^ These optimized deposition conditions ensure the fabrication of phase-pure and high-crystallinity CuCrO_2_ thin films suitable for advanced applications.

**Table 2:** Various Phases of CuCrO_2_ as Analyzed Using XRD

S·NO	Target type	Sputtering ower and Gas flow	2θ value	Secondary phases formed	Reference
1	Cu_2_O and Cr_2_O_3_	RF: 50, 200 W 10 sccm	CuO: 38°, 57°	CuO, Cr_2_O_3_	^40^
			Cr_2_O_3_: 33°, 54°		
2	CuCr alloy target	DC:200 W	CuO: 35°,38°	CuO, CuCr_2_O_4_	^61^
		O_2_/(Ar + O_2_) = 70%	CuCr_2_O_4_: 30°		
3	CuCrO_2_ ceramic target	RF: 150 W			^62^
		Ar/O_2_: 30/8 sccm			
4	ceramic CCO target (Cu/Cr = 1:1 ratio)	RF:150 W	CrN: 37.6°	CrN, CuN_3_	^50^
		Ar/N_2_:1 Pa	CuN_3_: 38.4°		
5	ceramic target	RF:150 W	CuCr_2_O_4:_ 57°,61.5°	CuCr_2_O_4_	^44^

Furthermore, postdeposition annealing plays a critical role in enhancing the crystal quality of CuCrO_2_ thin films and reducing secondary phases. Several studies discussed in this section have utilized postdeposition annealing in the temperature range of 900–1000 °C. This high-temperature treatment facilitates the removal of residual stresses, impurities, and secondary phases, thus promoting the formation of the delafossite phase and improving the crystallinity of the films. By annealing at such elevated temperatures, secondary phases such as CuO and CuCr_2_O_4_ are minimized, leading to stabilization of the desired delafossite structure. This annealing process is essential for achieving high-performance CuCrO_2_ thin films with improved structural integrity, as demonstrated in the work discussed here.

#### 2.1.4 Raman Spectroscopy

Raman spectroscopy is a nondestructive vibrational characterization technique that provides insights into the bonding and crystal structure of materials. It is based on the inelastic scattering of monochromatic light, usually from a visible- or near-infrared laser source. When incident photons interact with the lattice, most are elastically scattered without a change in energy (Rayleigh scattering). A small fraction, however, exchanges energy with vibrational modes of the lattice (phonons), resulting in a shift in photon energy. This phenomenon, known as Raman scattering, produces spectral features that correspond to the specific vibrational modes of the material. The Raman spectrum is typically plotted as intensity versus wavenumber shift (cm^–1^), measured relative to the excitation source. Because these vibrational modes are directly linked to the chemical bonding and symmetry of the crystal lattice, Raman spectroscopy provides a fingerprint unique to each phase. It is therefore widely used to confirm phase formation, assess crystallinity, and detect secondary phases and structural distortions. Compared with X-ray diffraction, which probes long-range order, Raman spectroscopy is especially sensitive to local bonding environments, making it a powerful complementary tool for thin-film analysis.

In the case of CuCrO_2_ thin films, Raman spectroscopy is particularly useful for identifying the characteristic vibrational modes of the delafossite phase and distinguishing it from possible secondary phases such as CuO, Cu_2_O, or Cr_2_O_3_. Each of these phases has distinct Raman signatures, and the presence or absence of their characteristic peaks provides direct evidence of the phase purity. This makes Raman a highly sensitive technique for confirming whether the sputtering and annealing conditions successfully stabilize the CuCrO_2_ structure or lead to competing oxides. Beyond simple phase identification, Raman spectroscopy also probes the local bonding environment within the Cu–O and Cr–O sublattices. Shifts in peak position, broadening of spectral features, or changes in relative intensity can indicate variations in crystallinity, strain within the lattice, or the presence of structural defects, such as oxygen vacancies. These vibrational fingerprints reflect how atoms are bonded and arranged locally in the lattice, offering information that complements diffraction techniques, which primarily measure the long-range order. For CuCrO_2_ specifically, Raman analysis is especially valuable because the delafossite phase often forms in competition with other copper and chromium oxides. By direct probing of lattice vibrations, Raman spectroscopy verifies the integrity of the Cu–O and Cr–O bonds that define the delafossite structure. As such, it is a powerful tool for characterizing thin films during optimization of deposition and annealing conditions, ensuring phase purity, and correlating microstructural quality with functional performance.^64^

The primitive unit cell of CuCrO_2_ (space group *R*_*m*_) consists of 4 atoms, leading to 12 normal modes of vibration. These are represented as Γ = a_1g_ + E_g_+3a_2u_+3E_u_. E_g_ is related to vibrations on a triangular plane perpendicular to the *c*-axis and A_1g_ is associated with Cu–O bond vibrations along the *c*-axis.^65^

The Raman spectrum of the sputtered CuCrO_2_ thin films provides significant evidence of its structural integrity. Ahmadi et al.·observed characteristic Raman peaks at 207, 441, and 668 cm^–1^, which correspond to distinct vibrational modes indicative of the delafossite phase with a rhombohedral crystal structure which can be observed in Figure 11. The confirmation of these peaks strongly supports the successful formation of sputtered CuCrO_2_, validating its suitability for optoelectronic applications. Furthermore, in heterostructure-based device configurations, CuCrO_2_ is often paired with n-type materials such as Al-doped ZnO (AZO). The Raman spectra of AZO exhibit characteristic vibrational peaks at approximately 277, 375, 439, 510, 580, and 645 cm^–1^, corresponding to various optical phonon modes including transverse optical and longitudinal optical vibrations. These Raman signatures confirm the formation of a hexagonal AZO phase, which complements the rhombohedral CuCrO_2_ structure in heterojunction-based optoelectronic devices. The precise identification of Raman-active modes in sputtered CuCrO_2_ films is crucial for assessing crystallinity, phase purity, and defect states, directly impacting the material’s performance in photovoltaic and photodetector applications.^45^

**Figure 11:**
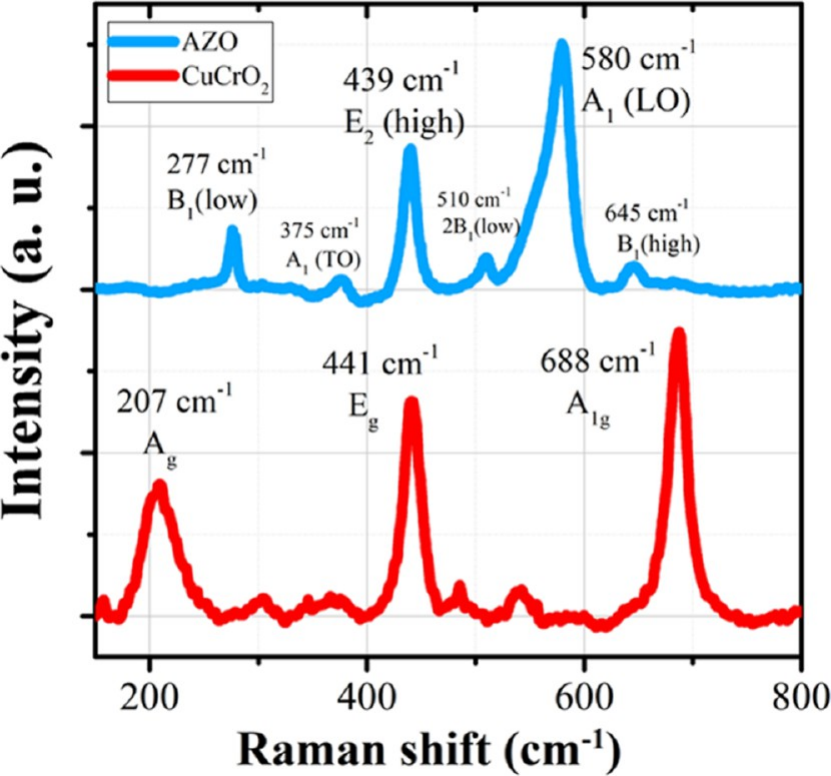
Raman scattering spectra of the deposited AZO and codoped CuCrO_2_ layers. Reprinted under the terms of the Creative Commons (CC BY-4.0) license.^45^

Garg and Rao et al.^66^ conducted Raman spectroscopic analysis to investigate the high-pressure behavior of copper delafossites, focusing on vibrational modes associated with Cu–O and M–O bonds (where M is a trivalent metal cation). Raman spectra confirmed the rhombohedral symmetry and single-phase nature of the samples, with two prominent modes identified: the A_1_g mode, corresponding to vibrations along the *c*-axis, and the E_g_ mode, corresponding to vibrations perpendicular to the *c*-axis (Figure 12). The ionic radius of the trivalent cation was found to exert a significant influence on these vibrational features. As the ionic radius decreased from La^3+^ to Cr^3+^, both A_1g_ and E_g_ modes shifted systematically to higher frequencies, a trend attributed to lattice contraction. This interpretation was reinforced by ab initio calculations as well as polarized Raman measurements, both of which confirmed the sensitivity of the vibrational modes to changes in the lattice dimensions. Substitution studies further demonstrated that cation size directly governed Raman mode frequencies, with the E_g_ mode being especially sensitive to variations in ionic radius.

**Figure 12:**
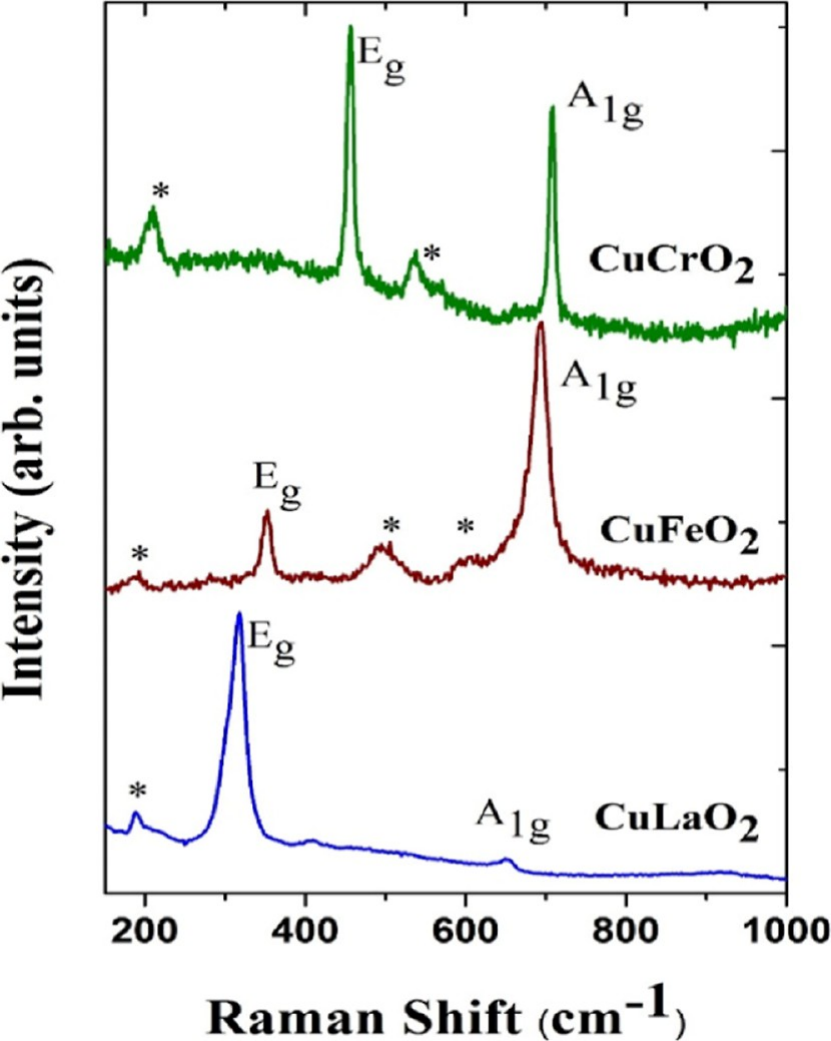
Raman spectra of as-synthesized CuLaO_2_, CuFeO_2_, and CuCrO_2_, showing two allowed Raman modes, a few weak modes shown by asterisks are disorder-induced nonzone center modes. Reprinted under the terms of the Creative Commons (CC BY-4.0) license.^66^

In doped CuCrO_2_, lattice parameters followed Vegard’s law, confirming systematic variation with dopant concentration. However, Raman spectra revealed additional broadening of the vibrational peaks, consistent with the introduction of local lattice distortions. For example, scandium substitution in both CuCrO_2_ and CuFeO_2_ was observed to soften Raman modes, indicating enhanced lattice disorder and phonon scattering effects. Complementary temperature-dependent Raman studies on CuFeO_2_ further revealed mode softening at elevated temperatures, attributed to thermal lattice expansion and phonon–phonon interactions. Taken together, these findings demonstrate that external parameters such as pressure, ionic radius, doping, and temperature strongly govern the vibrational properties of delafossites. Raman spectroscopy thus provides crucial insights into their structural responses under varying physical conditions, bridging experimental evidence with theoretical predictions.^66^

The study by Chiba et al. examines the optical and structural properties of CuCrO_2_ thin films deposited on c-face sapphire substrates using reactive RF magnetron sputtering. Raman spectroscopy confirms the presence of characteristic vibrational modes associated with the *R*3̅*m* space group, with observed peaks at ∼203 cm^–1^ (A_g_), 450 cm^–1^ (E_g_), and 705 cm^–1^ (A_1g_), indicating strong phase formation as observed in Figure 13. The unexpected A_g_ peak suggests a relaxation of Raman selection rules due to crystal orientation or intrinsic defects such as Cu vacancies and oxygen interstitials. The study also highlights the impact of nitrogen incorporation on the Raman intensity, with an optimal N_2_ flow ratio (∼10%) enhancing O–Cu–O bond formation by suppressing CuO, whereas excessive nitrogen reduces in-plane symmetry and deteriorates film quality.^52^

**Figure 13:**
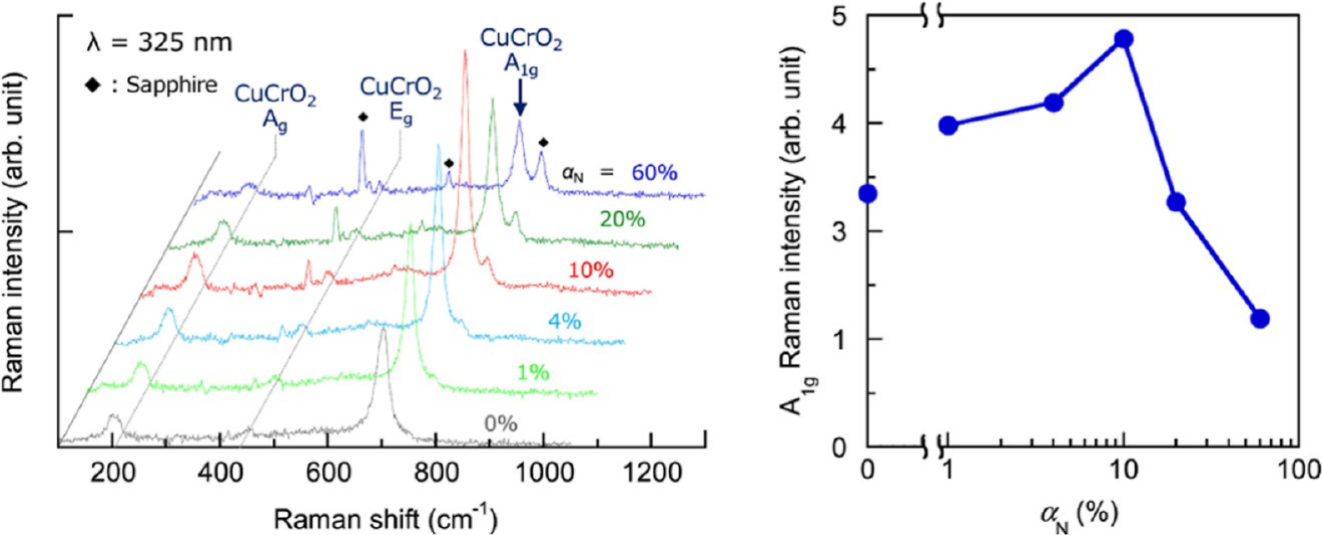
Raman spectra and dependence of A1g Raman integral intensity on αN. Copyright, Elsevier 2018.

In conclusion, Raman analysis of CuCrO_2_ thin films deposited via sputtering has provided valuable insights into the vibrational properties of the material. Table 3 summarizes all observed Raman modes and their corresponding shifts, highlighting the spectral variations that occur under different sputtering parameters and postdeposition treatments. The intensity and positions of the Raman modes are influenced by several factors, including sputter deposition parameters and postdeposition annealing. As discussed, annealing in a vacuum environment plays a crucial role in removing impurities and secondary modes in the spectrum, thus improving the overall quality of the films. This is in agreement with findings in ref 45 where postdeposition annealing at 900 °C for 2 h effectively facilitated the removal of secondary phases and helped in obtaining the desired delafossite phase. The sputtering technique not only allows for precise control over film deposition but also contributes to achieving high-quality CuCrO_2_ thin films by promoting the formation of the delafossite structure while reducing the number of undesired secondary phases. The findings from Raman spectroscopy underscore the importance of optimization in sputtering conditions and annealing processes to enhance the properties and performance of CuCrO_2_ thin films for various applications.

**Table 3:** Raman Modes in CuCrO_2_ Thin Films

Target type	Sputtering Power and Gas flow	Raman modes	Raman Shift (cm^–1^)	Excitation light wavelength	Reference
ceramic target	RF:150 W	A_1g_, E_g,_ A_g_	E_g_:441	785 nm	^45^
	Ar and N_2_: 0.12 mbar		A_1g_:688		
			A_g_:207		
ceramic target		_,_A_1g_,E_g_	A_1g_:700	325 nm	^66^
			E_g_:435		
ceramic target	RF:150 W	A_g_,E_g_,A_1g_	A_g_:203	325 nm	^52^
	Ar and N_2_: 1.0 Pa		E_g_:450		
			A_1g_:705		

The predominant Raman modes associated with CuCrO_2_ include the E_g_ mode at around 460 cm^–1^ and the A_1g_ mode at approximately 706 cm^–1^. These modes are typically observed and used to identify the presence of CuCrO_2_ in various studies.^13,52,67,68^

### 2.2 X-ray Photoelectron Spectroscopy Analysis

To understand the composition of the delafossite material, the usage of XPS which works on the principle of the photoelectric effect and the basic equation of the XPS can also be represented mathematically as^69^

1 $$E_{b}=hv-E_{k}$$

*E*_b_ is the electron binding energy, *E*_k_ is the kinetic energy of the electron measured by the instrument, and *hv* is the photon energy. Generally, the intensity of the core peak is related to several factors such as the intensity of the X-rays, the probability for X-ray/atomic orbital interaction also known as the photoionization cross-section. We can also give a generalization for the carrier concentration of any elements present in the compound.

2 $$N_{i,k}=I_{o}\rho_{i}\sigma_{i,k}\lambda_{i,k}T_{i,k}$$

where *k* is the *k*_th_ shell of an atom of type *I* in the sample, *I*_0_ is the X-ray flux incident on the sample, ρ is the volume density, σ is the differential photoionization cross-section for the *k* shell in the *i*th atom, and *T*_*i*,*k*_ is the transmission or throughput function at the kinetic energy of the electrons from the *k* shell. In the case of the material discussed here, CuCrO_2_ forms a delafossite structure comprising Cu, Cr, and O atoms, which have binding energies of Cu_2p_, Cr_2p_, and O_1s_. Unlike other structures, the delafossite structure requires a focus on the satellite mentioned above peaks, mainly found at lower energies, to distinguish between Cu^1+^ and Cu^2+,^ with Cu^1+^ corresponding to the delafossite structure’s formation. The Cu^1+^ satellite peaks appear at lower energies due to the metal–ligand charge transfer occurring in Cu^1+^, which ensures the stable formation of the delafossite compound.^70^ In the XPS spectra of Cu-based delafossites, the determination of copper oxidation states is typically performed through a detailed analysis of the Cu 2p core-level peaks and their associated satellite features. Cu^2+^ ions are characterized by the presence of prominent shakeup satellites located approximately 9–10 eV above the main 2p_3/2_ and 2p_1/2_ peaks, arising from strong metal–ligand charge–transfer interactions. In contrast, Cu^1+^ species generally exhibit sharper 2p peaks without pronounced satellite features, reflecting the filled 3d^10^ electronic configuration and weaker final state effects. The main Cu 2p_3/2_ binding energy for Cu^1+^ in delafossite structures typically lies in the range of 932.2–932.8 eV, whereas Cu^2+^ appears at slightly higher energies around 933.5–934.5 eV, providing a quantitative basis for oxidation-state identification.

The presence of Cu^1+^ is of particular significance for stabilizing the delafossite structure. In CuMO_2_ (M = Cr, Al, Ga, etc.), the Cu^1+^ ions occupy linear coordination sites between oxygen layers, maintaining charge balance and promoting the layered stacking of Cu–O and BO_2_ sheets. This arrangement is crucial for achieving phase-pure films and avoiding the formation of competing oxide phases, such as CuO or Cr_2_O_3_, which can otherwise compromise structural integrity. Moreover, the Cu^1+^ state directly influences the electronic structure, contributing to a dispersive valence band dominated by Cu 3d–O 2p hybridization, which in turn affects hole mobility and optical transparency. Therefore, XPS analysis of the Cu 2p region not only serves as a diagnostic tool for oxidation-state identification but also provides indirect insight into phase formation, crystallinity, and the electronic properties critical for device performance in transparent electronics and photovoltaic applications.

#### 2.2.1 Survey Scan Studies

The XPS survey spectra of CuCrO_2_ thin films, as presented by Sundaresh et al. in Figure 14, confirm the presence of only Cu, Cr, and O elements, emphasizing the compositional purity of the films. This purity is particularly critical for maintaining the delafossite structure, known for its unique electronic and optical properties attributed to the presence of the Cu^+^ species. A key feature distinguishing Cu^+^ in XPS is the absence of satellite peaks in the Cu-2p core-level spectra, in contrast to Cu^2+^, which exhibits shakeup satellite features due to metal–ligand charge transfer during photoemission. As demonstrated by this paper, such detailed surface characterization provides valuable insights into the properties of delafossite materials, reinforcing their potential for advanced functional applications.^41^

**Figure 14:**
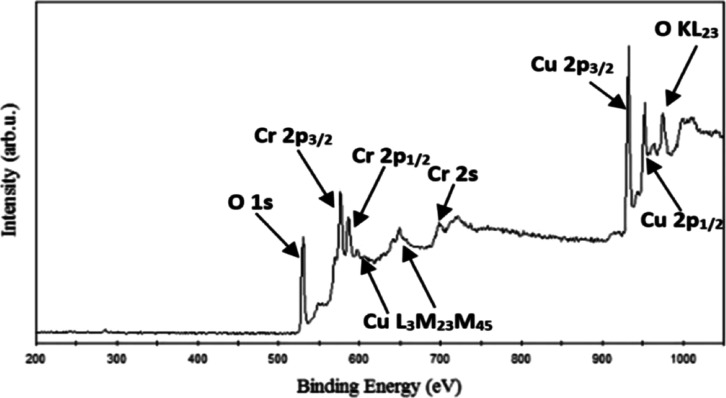
. XPS survey spectrum of CuCrO_2_ under the terms of Creative Commons (CC BY-NC-NC 3.0) license.

The X-ray Photoelectron Spectroscopy analysis of CuCrO_2_ thin films, as reported by Bharath et al., provides a detailed examination of their surface composition, focusing on the Cu 2p core-level spectrum (Figure 15). Peaks observed at binding energies of 951.2 eV (Cu 2p_1_/_2_) and 931.4 eV (Cu 2p_3_/_2_) confirm the presence of Cu^+^ species, which are characteristic of the delafossite CuCrO_2_ phase. Notably, the absence of satellite peaks in the 940–950 eV range distinctly indicates the absence of Cu^2+^ species typically found in the spinel CuCr_2_O_4_ phase. The O 1s spectrum supports these findings with peaks at 529.05 eV, associated with lattice oxygen, and peaks at 530.53 eV, attributed to chemisorbed oxygen, further validating the oxidation states of Cu^+^ and Cr^3+^ in the delafossite structure. This surface-level analysis emphasizes the high compositional purity and successful synthesis of single-phase CuCrO_2_ thin films, underlining their potential for advanced applications.^40^

**Figure 15:**
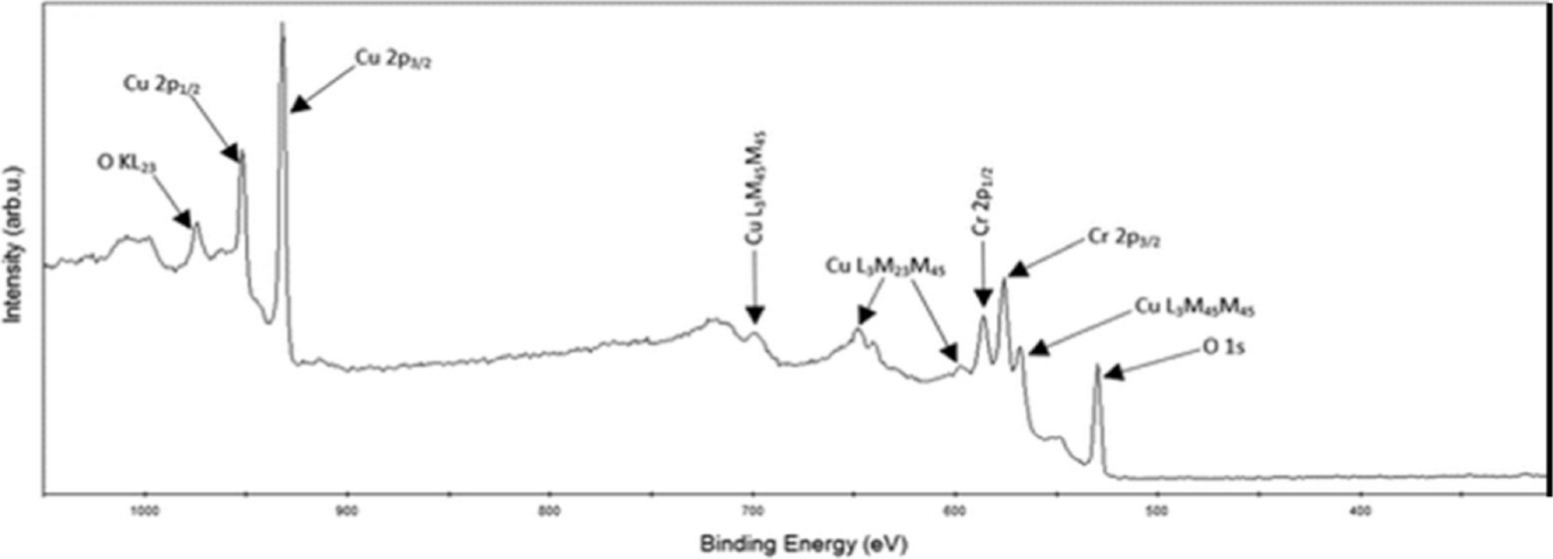
XPS spectra of the CuCrO_2_ film deposited with a copper sputtering power of 20 W under the terms of Creative Commons (CC BY-NC3.0) license.

The XPS survey scan of CuCrO_2_ thin films confirmed that copper, chromium, and oxygen are the primary constituent elements with no significant contamination detected. A more detailed examination of the Cu 2p core-level region revealed contributions from both the Cu^+^ and Cu^2+^ oxidation states. The distinction between these states is primarily based on the presence or absence of shakeup satellite peaks: Cu^2+^ exhibits intense satellite features approximately 9–10 eV above the main 2p_3/2_ and 2p_1/2_ peaks, which arise from strong metal–ligand charge–transfer interactions and characteristic final-state effects. In contrast, Cu^+^ displays sharp, well-defined 2p peaks without pronounced satellites, consistent with its filled 3d^10^ electronic configuration and weaker final-state interactions. Quantitatively, the Cu 2p_3/2_ binding energy for Cu^+^ is typically observed around 932–933 eV, while Cu^2+^ appears at slightly higher energies (∼933–934 eV), providing a reliable basis for oxidation-state identification.

The prevalence of Cu^+^ in the CuCrO_2_ thin films is particularly significant as it plays a critical structural and electronic role in stabilizing the delafossite lattice. Cu^+^ ions occupy linear coordination sites between oxygen layers, maintaining charge balance and promoting the characteristic layered stacking of Cu–O and CrO_2_ sheets. This structural arrangement is essential for achieving phase-pure films and preventing the formation of secondary oxides such as CuO and Cr_2_O_3_, which could compromise crystallinity, electronic transport, and optical properties. Furthermore, the Cu^+^ state contributes to a dispersive valence band through strong Cu 3d–O 2p hybridization, enhancing hole mobility and ensuring the desired optoelectronic performance.

Thus, the XPS oxidation-state analysis not only verifies the elemental composition of CuCrO_2_ thin films but also provides critical insight into the intrinsic role of Cu^+^ in maintaining the structural integrity, phase stability, and functional properties. This underscores the importance of carefully controlling deposition and annealing conditions to maximize the Cu^+^ content and to optimize the performance of delafossite-based thin-film devices.

#### 2.2.2 Core-Level Analysis

By analyzing the Cu 2p, Cr 2p, and O 1s core levels, X-ray photoelectron spectroscopy reveals important information on the oxidation state and local coordination for CuCrO_2_ thin films, as depicted in Figure 16a–c. The Cu 2p core-level spectrum (Figure 16a) shows the peaks at the binding energies of 951.2 and 931.4 eV are used, which refer to Cu 2p_1/2_ and Cu 2p_3/2_, respectively, and no satellite peaks are observed in the 940–950 eV range. Singularly missing from these bands are satellite peaks that are normally associated with charge transfer processes originating from Cu^2+^ ions, hence verifying that copper is indeed in the +1 state with minimal interruption of the lattice by oxygen vacancies. The Cr 2p spectrum (Figure 16b) shows that two peaks at 585.06 and 575.04 eV correspond to Cr 2p_1/2_ and Cr 2p_3/2_, respectively, which suggests that chromium exists in the +3-oxidation state. In the O 1s spectrum (Figure 16c), the first peak at 529.05 eV is assigned to the lattice oxygen in CuCrO_2_, while the second peak at 530.53 eV is possibly due to the chemisorbed oxygen from oxygen vacancies or surface hydroxyl groups. The binding energies and peak positions agree with the results from the literature, indicating that the synthesis of pure-phase delafossite CuCrO_2_ is successful. There are no Cu^2+^ satellite features and increased lattice oxygen intensity to emphasize that the CuCrO_2_ composition remains stoichiometric and, through proper synthesis, oxygen vacancies are kept to a minimum.

**Figure 16:**
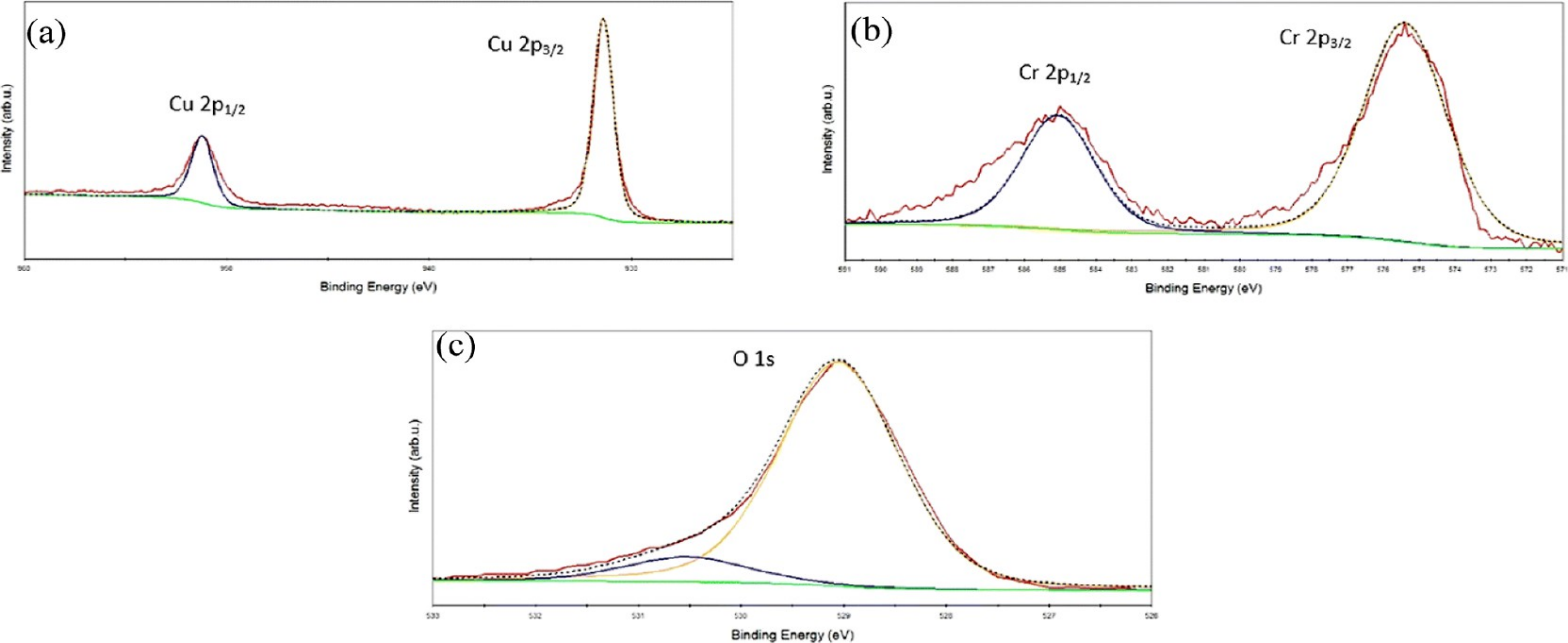
Core-level analysis of (a) Cu, (b) Cr, and (c) O_2_. Reprinted under the terms of Creative Commons (CC BY-NC3.0)^41^ license.

The XPS spectra of the CuCrO_2_ thin films, giving X-ray photoelectron spectroscopy analysis in [Figure 17a–e­(i,iii)], contain very important information about the oxidation states of the CuCrO_2_ films and activating the role of oxygen deficiencies for the appearance of satellite peaks in the Cu 2p spectrum. At 10 W sputtering power, we were unable to identify any Cu peaks on the film surface, while there may be CuCr_2_O_4_ phases below the surface. This is because XPS, being a surface-sensitive analytical tool, cannot identify buried species. The appearance of Cu 2p_3_/_2_ and Cu 2p_1_/_2_ at 30 W [Figure 17b (i)] and weak satellite peaks at higher BE also approve the Cu^2+^ species. These satellite peaks are due to charge transfer effects from unpaired d-electrons in Cu^2+^, mediated by oxygens vacancies, which unstabilize the Cu^+^ state enabling partial oxidation of Cu^+^ to Cu^2+^. This results in the satellite peaks at 50 W sputtering power [Figure 17(c,i)] being reduced, showing that the Cu^+^ ions are stabilized by suppressing oxygen vacancies. This corresponds to the formation of a stoichiometric, single-phase CuCrO_2_ delafossite structure in which Cu is divalent Cu^+1^. However, as the sputtering power went higher to 75 and 100 W, the satellite peaks show up again in [Figure 17d,e (i)], which meant the Cu^2+^ species reemerged due to increase in oxygen vacancy, therefore causing the CuO phase formation due to nonstoichiometric structure. Consumed in Cr 2p spectra [Figure 17a–e (ii,iii)], Cr reveals its presence in the +3 oxidation state in all sorts of the films, while Cu LMM peak, signifying Cu^+^, is only noticeable at 50 W. Peculiarities in the O 1s region are shown in [Figure 17a–e,(iii)], and the analysis of the spectra revealed that there were less chemisorbed oxygen at 50 W due to the reduced vacancies in the lattice. Altogether, these outcomes suggest that oxygen vacancies are the cause of Cu-oxidation, and the pure CuCrO_2_ configuration is feasible only when 50 W is applied.^41^

**Figure 17:**
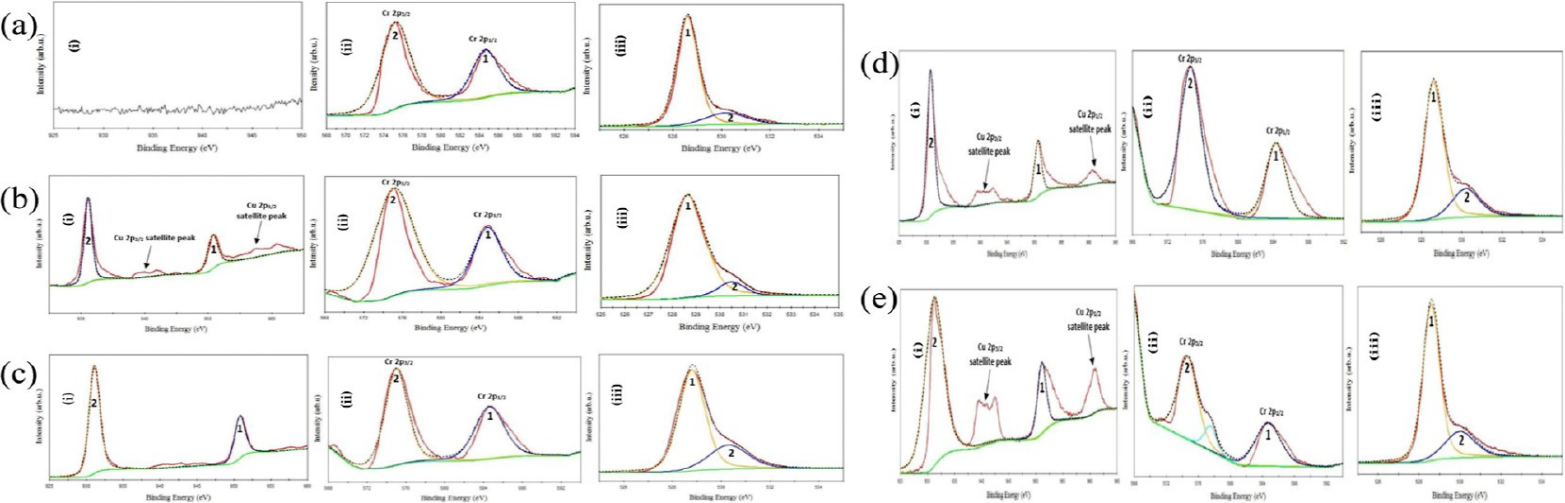
(i) Cu-2p core-level spectrum, (ii) Cr-2p core-level spectrum, (iii) O-1s core-level spectrum of thin films sputtered with (a) 10 W, (b) 30 W, (c) 50 W, (d) 75 W, and (e) 100 W of CuCrO_2_ sputtering power. Reprinted under the terms of Creative Commons (CC BY-NC-NC 3.0) license.^41^

X-ray Photoelectron Spectroscopy studies on CuCrO_2_ thin films have provided crucial insights into the elemental composition and oxidation states, which directly influence the formation of the delafossite phase. The findings indicate that the relative oxygen content in films is strongly affected by the Cu and Cr components. When the Cu content is significantly higher, with a Cu–Cr content discrepancy of 3.9 atomic percentage or more, the films tend to form a composite phase structure consisting of CuO and CuCrO_2_. This suggests that after CuCrO_2_ formation, excess Cu reacts with oxygen, leading to increased oxygen content in composite phase films compared to single-phase films. In contrast, the formation of single-phase CuCrO_2_ requires a composition ratio close to 1:1:2 (Cu/Cr/O), with a Cu–Cr content discrepancy of 2.6 at % or lower.^59^ The single-phase CuCrO_2_ films exhibit a nonstoichiometric nature due to an excess of oxygen and a deficiency of Cu and Cr, with O/Cu and O/Cr ratios ranging between 2.05 and 2.46. This excess oxygen plays a critical role in stabilizing the delafossite structure and serves as a key factor in hole carrier generation as hole carriers are primarily introduced by oxygen interstitials and vacancies of Cu and Cr. It is evident from these XPS studies that the formation of CuCrO_2_ in the delafossite phase strongly depends on optimizing the oxygen flow during sputtering, irrespective of the specific sputtering technique, target composition, or deposition parameters used. If the Cr content exceeds the required stoichiometric ratio, secondary phases such as CuCr_2_O_4_ or Cr_2_O_3_ emerge, which significantly affects the electrical properties of the films. Therefore, precise control over the Cu:Cr:O ratio at 1:1:2 is essential to achieving phase-pure CuCrO_2_ with optimal structural and electrical characteristics.^59^

### 2.3 Optical Studies

#### 2.3.1 UV–visible Spectroscopy

UV–visible spectroscopy is a frequently employed technique for assessing the absorption and transmission of both ultraviolet and visible light by a material.^71^ UV–vis spectroscopy is particularly useful in the study of CuCrO_2_ for showing changes in optical characteristics due to doping, analyzing electronic transitions, and calculating the bandgap. CuCrO_2_ has a usually low transmittance that varies depending on sputtering factors, such as the annealing temperature and gas flow ratio. Gaining these insights is crucial to optimizing CuCrO_2_ for electrical devices, sensors, and catalytic applications.

Chung-Hsing Sun et al. studied the optical properties of CuCrO_2_ films annealed at 800 °C for different annealing durations. The as-deposited film showed a transmittance of about 39.1% at a wavelength of 740 nm as shown in Figure 18. When annealed at 800 °C, transmittance significantly increased from 73.0% to 80.2% at the same wavelength as the annealing time increased from 30 to 240 min. This increase in transmittance and sharper absorption edge behavior are attributed to the growth of CuCrO_2_ crystals and the low defect density of the film. The optical direct bandgap of the thin films was revealed by Tauc’s plot relation, which decreased from 3.17 to 2.99 eV with the increase in annealing time. The as-deposited film was amorphous in nature and did not possess a direct energy gap. The optical bandgap behavior of the annealed CuCrO_2_ film aligns with the variation of grain size with annealing time, where annealing time increases the grain size, decreases the strain energy of the grain in the film, reduces quantum confinement, and results in lower optical bandgap.^55^

**Figure 18:**
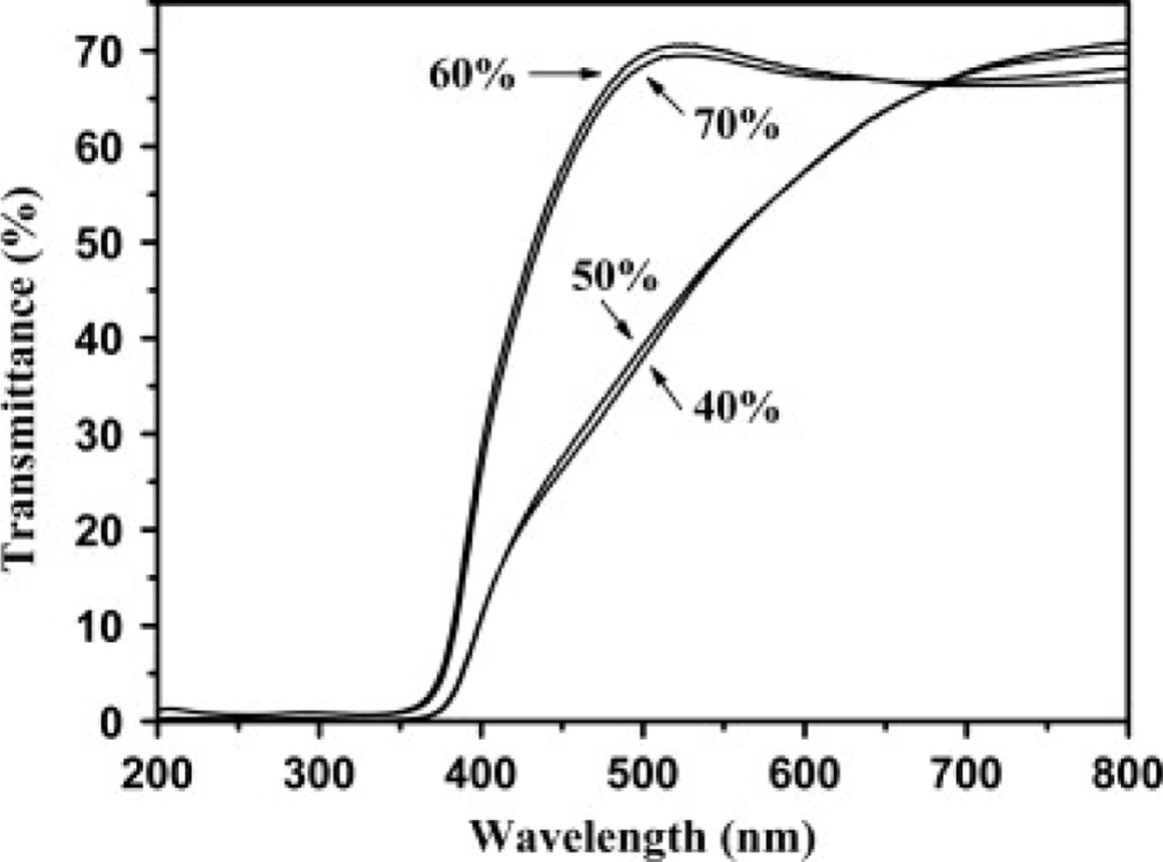
CuCrO_2_ thin films deposited at different O_2_/(O_2_+Ar) gas mass flow ratios. Reproduced with permission from ref 59. Copyright 2014, Elsevier.

The studies of Ruei-Sung Yun and Chorng-Pyng Tasi on the optical characteristics of p-type CuCrO_2_ is shown in Figure 19. Films with composite phases, comprising CuCrO_2_ and CuO and fabricated under different O_2_/(O_2_+Ar) gas mass flow ratios (40% and 50%), exhibited approximately 48% transmittance at a visible wavelength of 550 nm. On the other hand, single-phase CuCrO_2_ films produced at O_2_/(O_2_+Ar) ratios of 60% and 70% showed an increased transmittance of 68% at the same wavelength, representing a notable enhancement of approximately 20%. The transition from composite to single-phase films yielded two significant outcomes. First, the initial transmittance showed a blue shift. Second, the transmittance–wavelength relationship exhibited a steeper slope. The bandgaps of the composite-phase films were measured at 3.14 and 3.15 eV, respectively (shown in Figure 19). In contrast, the single-phase CuCrO_2_ films displayed slightly higher bandgaps of 3.18 and 3.17 eV, respectively. This increase is attributed to the elimination of the lower-bandgap CuO phase during the transformation into single-phase CuCrO_2_. The plots for single-phase CuCrO_2_ films revealed distinct bending at higher incident photon energies due to sub-band electron transitions. This phenomenon occurs when an incident photon is absorbed by an electron in the sub-band, leading to a transition into the conduction band’s sub-band while leaving behind a hole in the valence band’s sub-band. However, these transitions do not significantly contribute to the conductivity of the film.^59^

**Figure 19:**
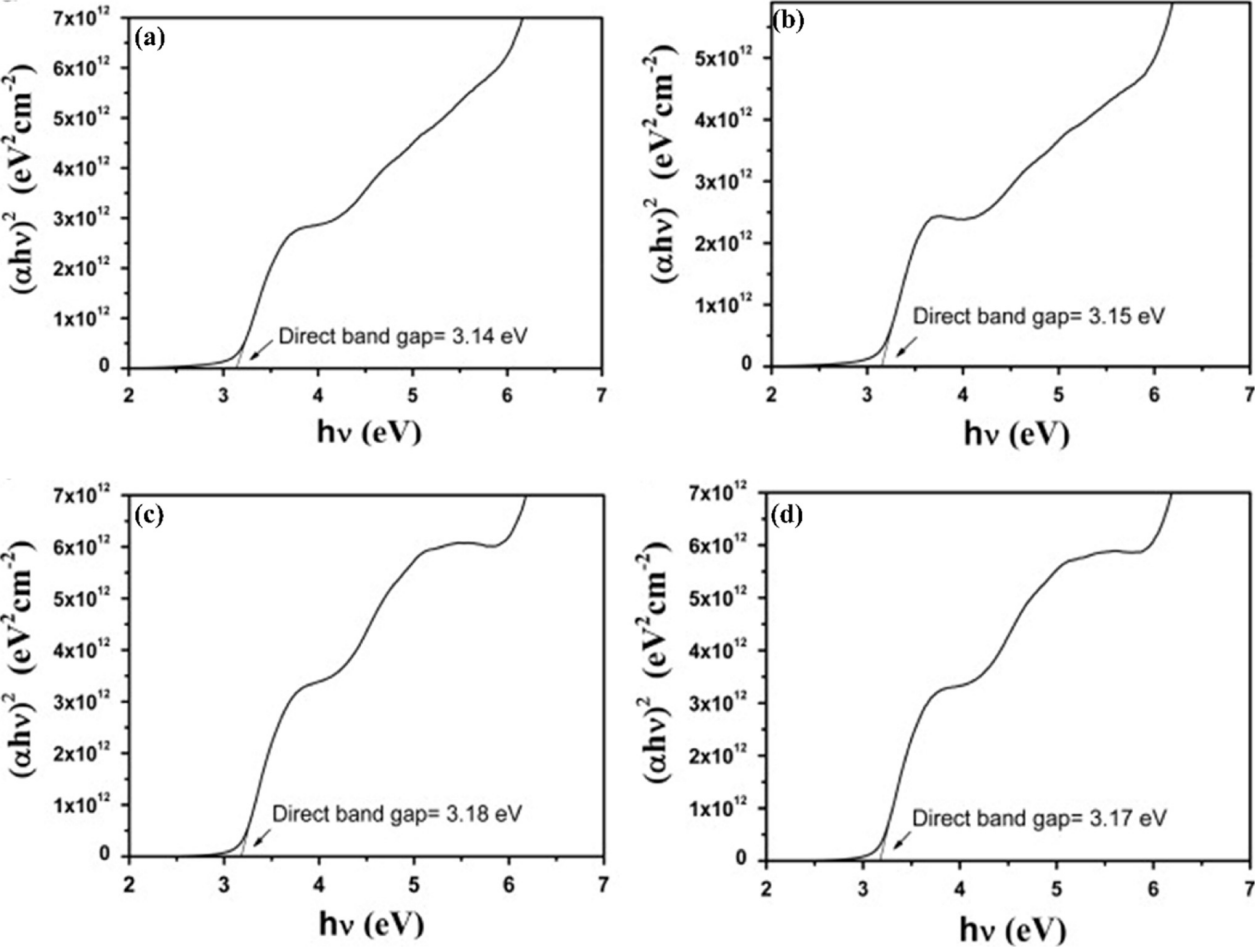
Tauc’s plot of CuCrO_2_ thin films prepared by deposition at O_2_/(O_2_+Ar) ratios (a) 40%, (b) 50%, (c) 60%, and (d) 70%. Reproduced with permission from ref 59. Copyright 2014, Elsevier.

Fuh-Sheng Shieu et al. investigated the optical characteristics of CuCrO_2_ films subjected to annealing at 700 °C, varying the power of the copper target (shown in Figure 20). Their findings revealed that the film produced at 22 W exhibited a distinct transition between absorption and transmission, indicative of a high-quality CuCrO_2_ phase. In contrast, films produced at significantly different powers displayed a more gradual transition, known as the absorption edge, likely due to impurities or defects. The study also observed that the 22 W film demonstrated the highest visible light transmittance of 58.31%, attributed to the narrow bandgaps characteristic of CuO and CuCr_2_O_4_, both of which are p-type semiconductors. However, with an increase in power to 52 W, the transmittance decreased to 17.74% due to inclusion of the monoclinic CuO phase. Moreover, escalating the Cu-target power led to a red shift in the absorption edge, indicating a shift from CuCr_2_O_4_ to CuCrO_2_ and an increase in the monoclinic CuO phase. The study acknowledged the challenge in directly measuring the energy gap due to the structural disorder or multiphase nature of the films. However, utilizing Tauc’s method, they estimated the direct bandgap of the CuCrO_2_ film at 22 W to be 3.18 eV, consistent with the existing literature. Finally, higher Cu-target power resulted in thicker films with greater absorption, leading to lower transmittance at shorter wavelengths.^48^ Te-Wei Chiu et al. also observed that the CuCrO_2_ films exhibit a high level of transmittance in the visible region, and as the film thickness reduces, the optical transmittance increases from 70% to 98%. The direct bandgap of CuCrO_2_ thin films, formed through a two-step annealing process, was determined to be 3.05 eV.^62^

**Figure 20:**
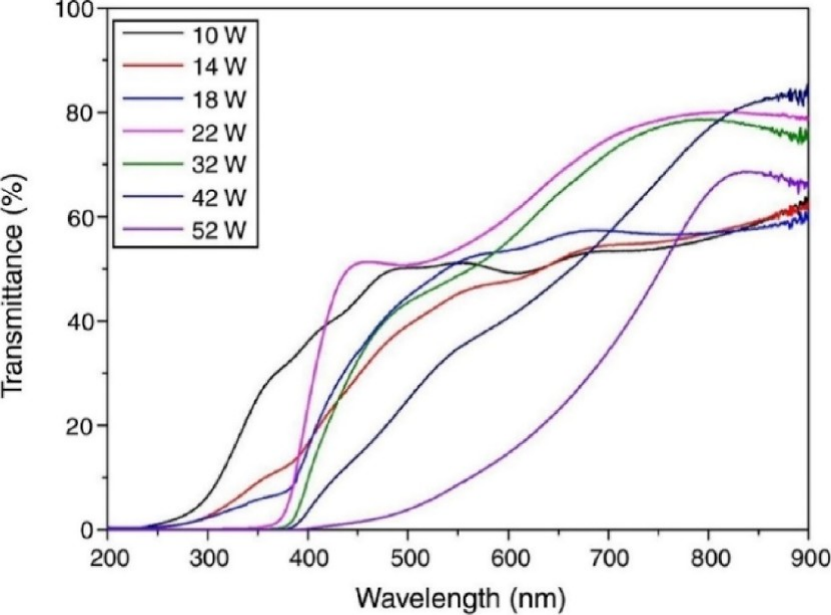
Transmittance spectra of the CuCrO_2_ coatings prepared at various target powers. Reproduced with permission from ref 48. Copyright 2019, Elsevier.

Ruei-Sung Yu et al. conducted a study on Cu–Cr–O films, specifically focusing on their light transmittance. Their investigation revealed that structural alterations within the films resulted in diverse light transmittance behaviors with each structural phase exhibiting a unique absorption coefficient. Notably, CuCrO_2_ films demonstrated a notable increase in transmittance at specific wavelengths compared to amorphous or two-phase structures. Films subjected to annealing at 600 and 625 °C displayed transmittance levels ranging between 62 and 67% across the 600–800 nm range, consistent with established values for CuCrO_2_. These values, however, can vary due to factors such as defects and film thickness. The absorption coefficient of these films exhibited a sharp rise at certain wavelengths, indicating inherent absorption characteristics and the presence of two distinct peaks in the spectra.^61^

From Table 4, the optical transmittance and the bandgap of CuCrO_2_ thin films can be seen, and they are influenced by a variety of factors such as film thickness, annealing time and temperature, O_2_/(O_2_+Ar) gas mass flow ratios, target power, and doping concentration. From these studies, an increase in the film’s thickness, annealing time, and target power results in a decrease in light transmittance and a reduction in the bandgap, which can be agreed for any thin film. Conversely, an increase in the O_2_/(O_2_+Ar) gas flow ratios, deposition and annealing temperature, leads to an increase in the transmittance of the CuCrO_2_ film, accompanied by a change in the bandgap which can range from 2.99 to 3.52 eV.

**Table 4:** Various Deposition Parameters of CuCrO_2_ and Their Effect on Optical Transmittance and Bandgap

Parameter			Transmittance (%)	Bandgap	Reference
thickness		280 nm	∼47% at ∼450 nm	3.23 to 3.13 eV	^53^
		70 nm	∼67% at ∼450 nm		
annealing time		As deposited	∼39.1% at 740 nm	3.17 to 2.99 eV	^55^
		30 min	∼73.0% at 740 nm		
		240 min	∼80.2% at 740 nm		
O_2_/(O_2_+Ar) gas flow ratios		40% and 50%	∼48% at 550 nm	3.14 and 3.15 eV	^62^
		60%and 70%	∼68% at 550 nm	3.18 and 3.17 eV	
varying copper target power		22 W	58.31% at visible region	3.18 eV	^59^
		52 W	17.74% at visible region		
annealing temperature (a-CuCrO_2_: N)		600 °C	45% at 700 nm		^41^
		650 °C	81% at 700 nm		
		750 °C	78% at 700 nm		
		800 °C	71% at 700 nm		
		900 °C	62% at 700 nm		
		600 °C	40.46% at visible region	3.52 eV	
		900 °C	48.57% at visible region	3.16 eV	
CuCrO_2_ and CuCrO_2_: N	undoped	as deposited	44%		^50^
		900 °C	67%		
		>550 °C		3.27 ± 0.2 eV	
	N_2_-doped	Up to 700 °C	increased		
		900 °C	63%		

#### 2.3.2 Photoluminescence Spectroscopy

Photoluminescence (PL) spectroscopy is an essential analytical technique used to study the light emission properties of materials when they are excited by photons. PL spectroscopy is invaluable for probing the electronic structure, defect states, and recombination mechanisms of the charge carriers. It provides insights into the effects of copper doping on the luminescent properties of chromium oxide, including the identification of defect levels and impurity states introduced by the doping process.^72^ The analysis of PL spectrum^43^ in CuCrO_2_ thin films provides valuable insights into their electronic and optical properties. Several studies have contributed significantly to this field, which helps us to understand the intricate behavior of defects and their impact on emission characteristics.

Ahmadi et al. studied the PL spectrum of codoped CuCrO_2_ thin films (shown in Figure 21). They observed a subtle peak at approximately 412 nm, which is indicative of band-to-band transitions in the sample. This peak, serving as a marker for the optical bandgap threshold, allowed them to estimate the bandgap of CuCrO_2_ to be around 3.01 eV. They also noted a broad and pronounced peak spanning the wavelengths from 450 to 550 nm, which is generally associated with defects in the CuCrO_2_ lattice. Additionally, another distinct peak was observed at 486 nm. This peak is believed to be due to copper deficiencies in the CuCrO_2_ lattice, which create trap states within the bandgap.^45^

**Figure 21:**
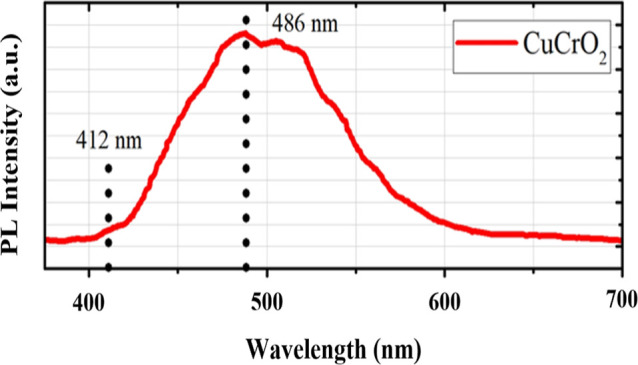
PL spectra of codoped CuCrO_2_ thin films. Reprinted from ref 42 under the terms of the Creative Commons (CC BY 4.0) license.

Ponmudi et al. also conducted a study on the PL spectra of CuCr_2_O_4_/CuCrO_4_ thin films (shown in Figure 22). These films were developed on quartz substrates and annealed at 1000 °C. The spectra revealed a distinct, intense emission peak at 530 nm (green), a broader medium-intensity peak at 509 nm (green), and a less pronounced peak at 475 nm (blue). These peaks were observed for films deposited at RF powers of 100, 200, and 300 W, respectively. The emission characteristics are likely due to structural irregularities and various defect states within the films, such as metal interstitials (Cr_i_ or Cu_i_), oxygen vacancies, metal vacancies (V_Cr_ or V_Cu_), and O_i_. As the RF power increases, the intensity of these peaks strengthens, and they shift toward longer wavelengths. This shift could indicate an increase in localized states within the film at higher RF power levels. Specifically, the PL peak intensity of the CuCr_2_O_4_/CuCrO_4_ film sputtered at 300 W increases notably and shows a red shift. This shift is likely due to morphological changes and an increase in the number of defective states. The prominent green emission peak at 530 nm primarily arises from emissions at defect levels associated with metal ion interstitials (Cr^3+^ and/or Cu^2+^) and singly ionized oxygen vacancies. As a result, the film fabricated at 300 W RF power displays a higher intensity of the green peak, potentially due to a greater number of singly ionized oxygen vacancies. These vacancies may serve as favorable sites for attracting NH_3_ molecules, making the 300 W sputtered CuCr_2_O_4_/CuCrO_4_ thin film a promising candidate for gas sensing applications.^73^

**Figure 22:**
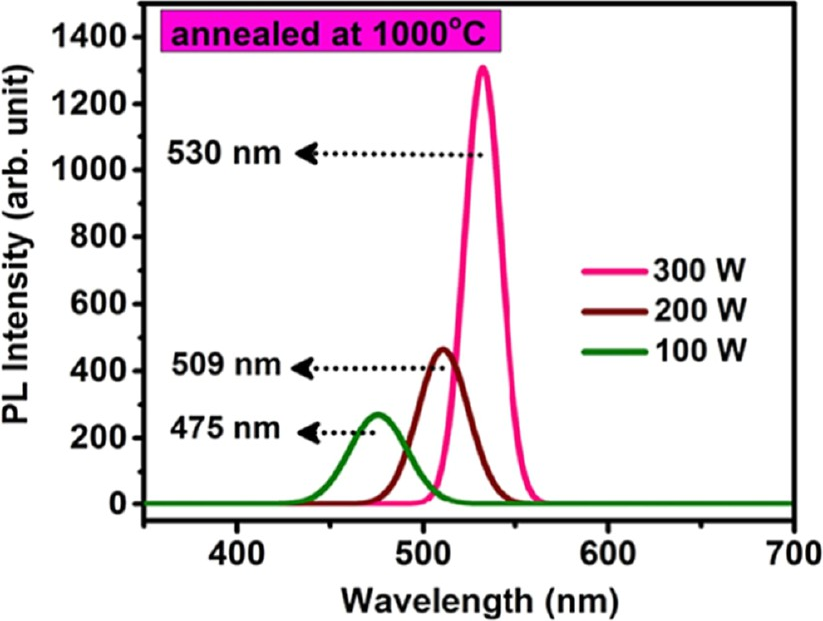
PL spectra of CuCr_2_O_4_/CuCrO_4_ thin films prepared on a quartz substrate. Reproduced with permission from ref 73. Copyright 2023, Elsevier.

Photoluminescence spectroscopy serves as a powerful tool for analyzing the optical properties and defect states in sputtered CuCrO_2_ thin films, aiding in the identification of the delafossite phase. The PL spectrum of CuCrO_2_ exhibits a distinct peak at ∼412 nm, corresponding to band-to-band transitions, allowing for an estimated bandgap of ∼3.01 eV.^62^ Additionally, a broad emission spanning 450–550 nm is attributed to defect states, particularly Cu vacancies and interstitial oxygen, which create localized trap states within the bandgap. The presence of these defect-related emissions confirms nonstoichiometric variations in oxygen content, which play a critical role in stabilizing the delafossite structure. Comparatively, CuCr_2_O_4_ /CuCrO_4_ thin films display distinct PL characteristics, including sharp emission peaks at 475, 509, and 530 nm, which are linked to metal interstitials and singly ionized oxygen vacancies.^73^ The broader defect-related emission in CuCrO_2_, as opposed to the well-defined peaks in CuCr_2_O_4_/CuCrO_4_, underscores the unique defect chemistry of the delafossite phase. These findings highlight the effectiveness of PL spectroscopy in distinguishing between different oxide phases and assessing the defect states critical to CuCrO_2_ thin film performance.

### 2.4 Surface Morphology Studies

The morphological characteristics of thin films can be studied by various characterization techniques, in which some distinct results are highlighted in this section. Scanning electron microscopy reveals surface features, whereas field emission scanning electron microscopy and field emission transmission electron microscopy analysis have enhanced resolution, allowing for precise visualization of surface morphology and identifying the defects in the thin films.^74–76^

#### 2.4.1 Scanning Electron Microscopy

This section discusses the morphology^53^ of the CuCrO_2_ thin films coated under different deposition and postprocessing conditions.

Sun, Hui, et al. studied the surface of CuCrO_2_ was studied using SEM imaging. They found that the surface structure was composed of particles, the side dimensions and form of which were contingent on the thickness, interspersed with gaps. As the thickness increased, the size of these gaps also expanded. Concurrently, the shape of the particles transitioned from spindle-shaped (shown in Figure 23a–c) to a faceted, plate-like form when the film’s thickness exceeded 210 nm (shown in Figure 23d). This transformation supports the increase in grain size with increasing thickness.^53^

**Figure 23:**
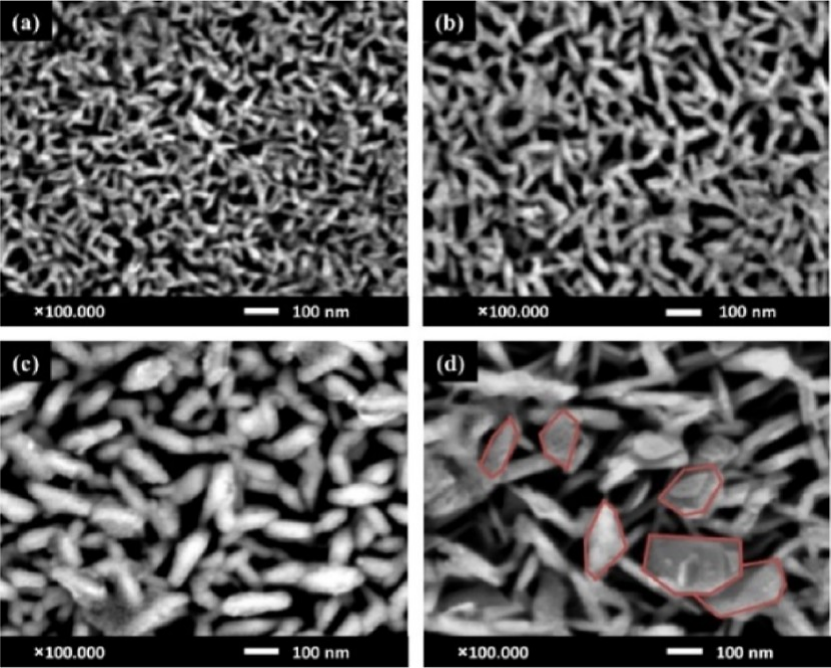
Surface SEM images of (a) CuCrO_2_ thin films with various thicknesses: (a) 70 nm, (b) 140 nm, (c) 210 nm, and (d) 280 nm. Reproduced with permission from ref 53. Copyright 2017, Elsevier.

Tsai, Du-Cheng, et al. conducted a study on the SEM images of CuCrO films annealed at 700 °C, with varying levels of Cu-target power as observed in Figure 24. Their observations revealed that as the Cu-target power increased from 10, 14, 18, 22, 32, 42, to 52 W, the film thickness correspondingly increased to 122, 133, 164, 167, 171, 212, and 315 nm. This increase in film thickness was attributed to the higher Cu-target power, supplying more Cu atoms to the growing films, thereby enhancing the deposition rate. The films prepared at a Cu-target power of 10 W exhibited a polygon-like surface with void boundaries, which was attributed to the formation of a crystalline phase. The growth of these voids was linked to volumetric changes associated with grain growth and phase transformations. When the Cu-target power was increased to 22 W, the film displayed significant grain growth and an increase in void size, indicative of pure CuCrO_2_ phase growth. At a Cu-target power of 42 W, the polygon-like clusters became smaller, suggesting the coexistence of CuCrO_2_ and CuO phases. However, further increasing the Cu-target power to 52 W led to the formation of larger polygon-like clusters, which could be attributed to the growth of the CuO phase.^48^

**Figure 24:**
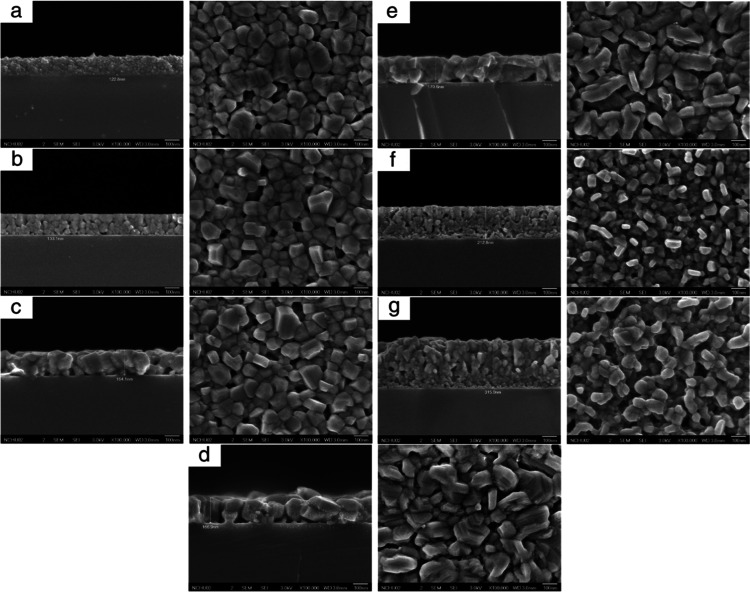
Cross-sectional and planar SEM images of the CuCrO_2_ coatings prepared at various Cu-target powers: (a) 10 W, (b) 14 W, (c) 18 W, (d) 22 W, (e) 32 W, (f) 42 W, and (g) 52 W. Reproduced with permission from ref 48. Copyright 2019, Elsevier.

Sun, Hui, et al. studied the cross-sectional SEM images of as-deposited and annealed (at 810 °C for 2 h under vacuum) CuCrO_2_ thin films deposited on an alumina substrate. The annealed thin film showed more porous (shown in Figure 25b) than the as-deposited film (shown in Figure 25a). This increased porosity is likely due to the volatilization of the Cu atoms at this high temperature. The volatilization can be accelerated in two ways: first, the continuous vacuum system reduces the partial pressure of Cu atoms on the film’s surface below their saturated vapor pressure at 810 °C, causing Cu atoms to migrate along paths with higher temperature gradients; second, the uniformly dispersed small Cu particles in the film have large surface areas, which facilitate their migration and volatilization. This also explains the formation of Cr_2_O_3_ in the annealed film.^27^

**Figure 25:**
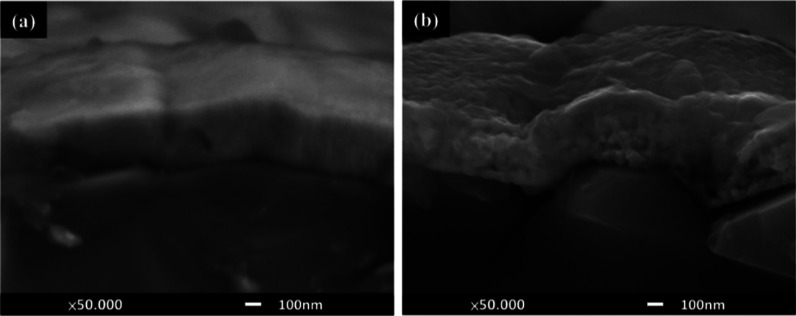
Cross-sectional SEM micrographs of the CuCrO_2_ thin film (a) as deposited and (b) after annealing. Reproduced with permission from ref 27. Copyright 2015, Elsevier.

#### 2.4.2 Field Emission Scanning Electron Microscope

Yu et al. studied the surface Field Emission Scanning Electron Microscope (FESEM) images as seen in Figure 26 of CuCrO_2_ thin films which were annealed for 3 min with varying temperature (as deposited, 550, 575, 600, and 625 °C). The image analysis of films annealed at 550 and 575 °C showed polygon-like particles on surfaces with voids. Before annealing, the films were amorphous with irregularly and loosely arranged Cu, Cr, and O atoms. Annealing caused atom diffusion, forming close-packed crystalline CuO and CuCr_2_O_4_, creating occasional voids. Films annealed at 600 and 625 °C displayed a surface with a mix of bar- and polygon-like features, attributed to the delafossite CuCrO_2_ structure formed during annealing. The 625 °C annealed film had larger surface features. The phase transformation is largely responsible for these changes in surface appearance.^61^

**Figure 26:**
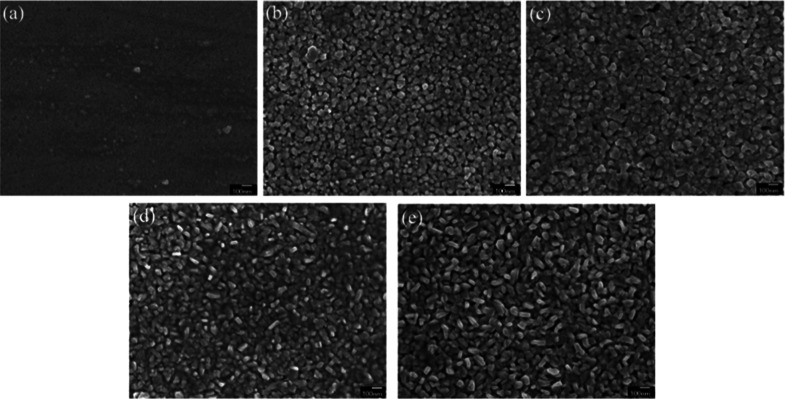
Surface FESEM images of CuCrO_2_ thin films (a) as deposited and annealed at (b) 550 °C, (c) 575 °C, (d) 600 °C, and (e) 625 °C. Reproduced with permission from ref 61. Copyright 2013, Elsevier.

A study by Sundaresh et al. examined the FESEM images of postdeposition annealed films at temperatures ranging from 600 to 900 °C, confirming nanocrystalline growth. The images in Figure 27a–e show that as the annealing temperature increases, the average grain size also increases, which grows from 40.22 nm at 600 °C to 105.31 nm at 900 °C. This growth occurs because higher thermal energy allows grains to coalesce. At temperatures 600 and 650 °C, the images show a smooth matrix without cracks. However, above 650 °C, gaps appear between grains due to increased thermal energy, consistent with previous studies. At 900 °C, rod-like structures characteristic of Cr_2_O_3_ nanostructures were observed along with the larger grains.^41^

**Figure 27:**
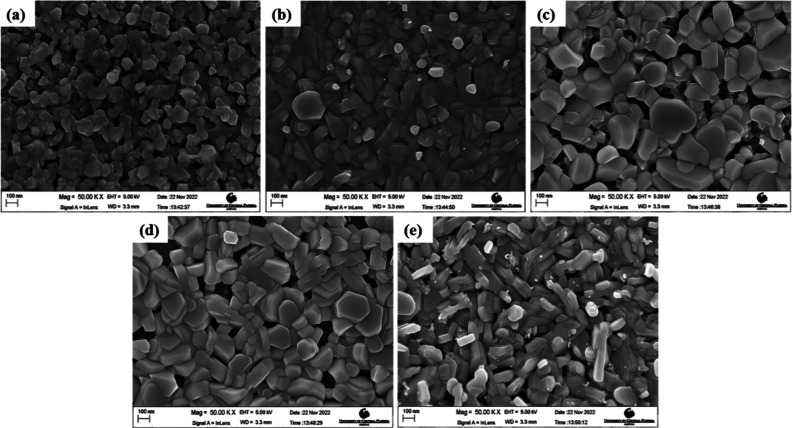
FESEM images of the films annealed at (a) 600 °C, (b) 650 °C, (c) 750 °C, (d) 800 °C, and (e) 900 °C. Reprinted from ref 41 under the terms of the Creative Commons (CC BY 4.0) license.

#### 2.4.3 Field Emission Transmission Electron Microscopy Analysis (FETEM)

Yu et al. studied the cross-sectional FETEM image of a sample containing quartz, CuCrO_2_, and polymer, as shown in Figure 28a. The CuCrO_2_ thin film exhibited a polygonal microstructure with a rolling surface, formed through annealing-induced phase transfer and atomic diffusion. Voids were observed near the interface between the film and the substrate. Figure 28b shows the nanoscale interface between quartz and the CuCrO_2_ thin film, revealing no compound phase formation due to high-temperature atomic interdiffusion. Figure 28c presents a high-resolution lattice image of CuCrO_2_, with a lattice spacing of 2.85 Å, identifying it as the (006) plane in the <006> direction.^59^

**Figure 28:**
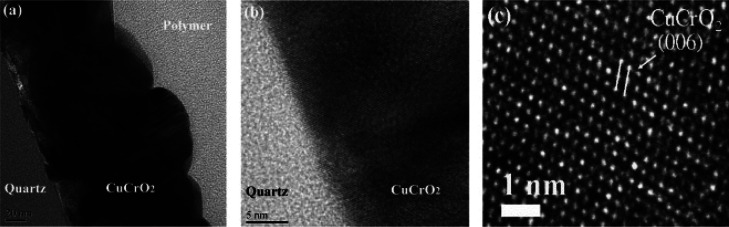
FETEM images of (a) CuCrO_2_ sample cross-section, (b) interface between quartz and CuCrO_2_ thin film, and (c) CuCrO_2_ (006) lattice plane reproduced with permission from ref 59. Copyright 2014, Elsevier.

Surface morphology studies reveal that increasing the Cu target power enhances deposition rates, resulting in thicker films. This increased thickness leads to expanded gaps and particle shape transitions with significant grain growth and phase transformations, forming CuCrO_2_ and CuO phases. Annealing at 810 °C increases film porosity, while annealing at 550–625 °C produces polygonal and bar-like particles, indicating phase changes to CuO and CuCr_2_O_4_. At higher temperatures (∼900 °C), grain size grows, causing gaps between grains and the formation of rod-like Cr_2_O_3_ structures. The CuCrO_2_ thin film develops a microstructure with voids near the substrate interface and no compound phase formation due to atomic interdiffusion at high temperatures.

### 2.5 Topological Analysis

#### 2.5.1 Atomic Force Microscopy

AFM is a powerful technique used to image and manipulate surfaces on the nanoscale, providing high-resolution topographical data. It operates by scanning a sharp probe across a surface and measuring the forces between the probe and the sample. One key metric derived from AFM data is the root-mean-square (RMS) roughness, which quantifies the average height variations of a surface relative to the mean plane. RMS roughness is calculated as the square root of the arithmetic mean of the squared deviations from the mean surface height, offering a robust measure of surface texture and uniformity.^77^

R. S. Yu et al. studied the surface topography and RMS roughness values of CuCrO films using AFM. The as-deposited amorphous film, as shown in Figure 29a, had a minimum RMS roughness value of 3.6 nm. However, annealing at 550 and 575 °C resulted in mixed CuO and CuCr_2_O_4_ phases, as shown in Figure 29b,c, and increased the RMS roughness values to 8.8 and 8.3 nm, respectively. Annealing at 600 and 625 °C, as shown in Figure 29d,e, resulted in a higher RMS roughness value that increased with the annealing temperature from 11.3 to 17.7 nm. The study also concluded that single-phase CuCrO_2_ film had the highest RMS roughness value.^61^

**Figure 29:**
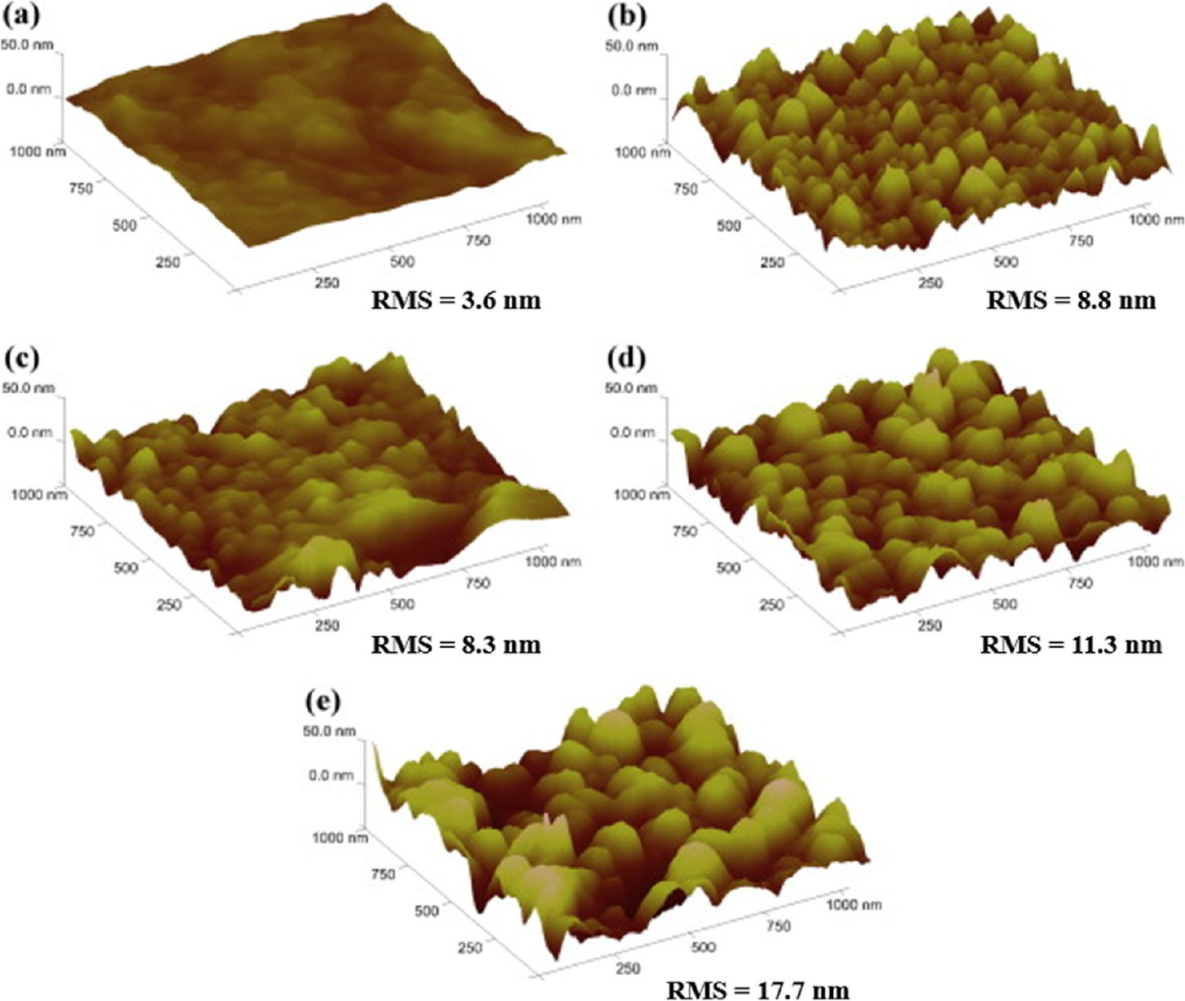
AFM surface topography (scale = 1 × 1 μm) images of (a) as-deposited film and films annealed at (b) 550 °C, (c) 575 °C, (d) 600 °C, and (e) 625 °C. Reproduced with permission from ref 61. Copyright 2013, Elsevier.

C. Y. Chen et al. conducted a surface topography study on CuCrO_2_ films deposited on Si and PMMA substrates (shown in Figure 30). The average RMS surface roughness of the CuCrO_2_ film on a Si substrate was 29.97 nm, with an average frequency cutoff (Ra) of 30.28 nm. In contrast, the CuCrO_2_ film deposited on a PMMA substrate had an average RMS roughness of 45.76 nm and an average Ra of 35.23 nm. The average radius of the CuCrO_2_ film was 83 nm on the Si substrate and 82 nm on the PMMA substrate. The CuCrO_2_ film on the Si substrate exhibited a smoother surface compared to the film on the PMMA substrate.^68^ Notably, the RMS roughness of the film annealed at 700 °C is slightly higher than that of the single-phase film annealed at 950 °C, likely due to the presence of mixed CuO and CuCr_2_O_4_ phases.

**Figure 30:**
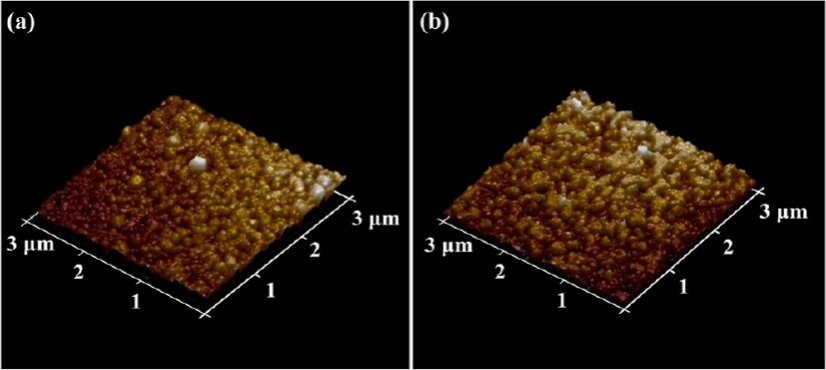
AFM surface topography (scale = 3 × 3 μm) images of the CuCrO_2_ film on (a) Si substrate and (b) PMMA substrate. Reproduced with permission from ref 68. Copyright 2021, Elsevier.

In conclusion, the roughness of as-deposited amorphous CuCrO_2_ films varied based on substrate type and annealing conditions. The minimum RMS roughness of 3.6 nm was observed in the as-deposited film. Annealing between 550 and 575 °C led to mixed CuO and CuCr_2_O_4_ phases, increasing the RMS roughness to 8.8 and 8.3 nm, respectively. Higher annealing temperatures of 600 and 625 °C, which formed CuCrO_2_, resulted in even greater roughness, ranging from 11.3 to 17.7 nm. The CuCrO_2_ films on Si and PMMA substrates showed distinct differences: the film on Si had an average RMS roughness of 29.97 nm and an Ra value of 30.28 nm, while the film on PMMA had a higher roughness with an average RMS of 45.76 nm and an Ra of 35.23 nm. Despite having similar average radii, the films on Si substrates were smoother than those on PMMA substrates. Improved crystallinity, particularly in films annealed up to 700 °C, was associated with increased RMS roughness due to the presence of mixed phases, slightly higher than those annealed at 950 °C.^68,78^

### 2.6 Electrical Studies

#### 2.6.1 Hall Effect Measurement

CuCrO_2_ is a p-type semiconductor belonging to the delafossite family, characterized by its layered structure and intrinsic hole conductivity. This material exhibits a relatively wide bandgap of approximately in the range of 2.9–3.5 eV, making it a promising candidate for various optoelectronic applications. The conduction mechanism in CuCrO_2_ is primarily governed by small polaron hopping, wherein charge transport occurs through localized hole states rather than delocalized band-like conduction. This unique transport behavior significantly impacts its electrical performance, necessitating a detailed investigation using Hall effect measurements.

Hall effect measurements play a crucial role in characterizing the electrical transport properties of CuCrO_2_ thin films by determining key parameters such as carrier concentration, mobility, and type of conductivity. Since CuCrO_2_ is inherently a p-type conductor, these measurements confirm the presence of holes as majority carriers. The Hall coefficient, obtained from these experiments, helps to quantify the charge carrier density, allowing researchers to understand the doping efficiency and how it influences electrical behavior. The carrier concentration in CuCrO_2_ thin films is highly dependent on factors such as synthesis techniques, deposition parameters, and postdeposition treatments. Doping and defect engineering further modulate these electrical properties, influencing the concentration of intrinsic and extrinsic carriers. Variations in Cu and Cr stoichiometry, oxygen partial pressure during deposition, and annealing conditions can either enhance or suppress carrier mobility. For instance, an optimal Cu/Cr/O ratio of 1:1:2 ^60^ is required to achieve a stable delafossite phase with minimal secondary phase formation. Deviations from this stoichiometry can introduce compensating defects such as oxygen vacancies or excess copper, which, in turn, affect the electrical properties.

Additionally, magnetotransport studies using Hall measurements provide deeper insights into the interaction between charge carriers and magnetic fields, further elucidating the role of defect states and their influence on carrier scattering mechanisms. Understanding these interactions is crucial for applications in spintronic devices, where control over the charge and spin transport is essential.

By systematically correlating electrical transport behavior with film synthesis and processing methods, researchers can tailor CuCrO_2_ thin films to meet specific application requirements. This tunability makes CuCrO_2_ highly relevant for emerging technologies including TCOs, thermoelectric devices, and optoelectronic components. A compiled table of the Hall Measurement is given in Table 5. From the table, it is evident that the carrier concentration of CuCrO_2_ thin films varies with different doping levels, directly influencing their electrical properties.^79^ Overall, the Hall effect serves as an indispensable tool in optimizing the electrical characteristics of CuCrO_2_ thin films, ensuring their suitability for next-generation electronic and optoelectronic applications.

**Table 5:** Electrical Properties of CuCrO_2_ and Doped CuCrO_2_ Thin Films Determined by Hall Effect Measurements

Thin film	Mobility (cm^2^ /(V s))	Carrier Concentration (cm ^-3^)	Resistivity (Ω cm)	Reference
CuCrO_2_	0.09	2.01 × 10^18^	34.72	^61^
Mg: CuCrO_2_	1.09 × 10^–3^	3.52 × 10^14^	4.08 × 10^–2^	^80^
CuCr_0.915_Zn_0.085_O_2_		1.67 × 10^17^	3.98	^81^
Cu (Cr_1–*x*_Fe_*x*_)O_2_		4.09 × 10^19^	0.036	^39^
CuCr_1–*x*_Ni_*x*_O_2_	0.18	4.93 × 10^18^	7.042	^82^
CuCr_1–*x*_Mg_*x*_O_2_	6.12	6.23 ×10^15^	50.77	^83^

## 3 Optimization of Magnetron Sputtering Parameters for High-Quality CuCrO_2_ Thin Films

This section outlines the key steps for achieving successful thin-film deposition of delafossite CuCrO_2_. Thin-film growth plays a crucial role in enabling heterostructures for device applications based on this material. While delafossite can be deposited using various techniques, we have specifically chosen sputtering to highlight its versatility and advantages, as underlined in previous sections. Before analyzing the deposition parameters, it is important to note that optimizing thin films is influenced by the type of sputtering (RF, DC, and pulsed DC) used, the precise handling and maintenance of the sputtering system, and its inherent effects on the performance of the system. Each of these elements plays a crucial role in determining the film quality, composition, and phase purity. The choice of sputtering method dictates energy transfer efficiency, ionization effects, and film growth dynamics, while meticulous system handling—such as target conditioning, substrate positioning, and vacuum integrity—ensures consistent deposition conditions. Additionally, factors such as chamber geometry, gas flow dynamics, and residual contamination can introduce secondary phases and defects in the thin-film properties. Hence, the following discussion focuses on the most critical parameters that can be fine-tuned to achieve phase-pure delafossite CuCrO_2_ thin films.

•Selection of suitable Sputtering target:

To perform sputtering and obtaining in-phase CuCrO_2_ delafossite, it is essential to understand the type of target material that is used. Ceramic targets such as CuCrO_2_, oxide targets like Cu_2_O and Cr_2_O_3_, powder targets including Cu/Cr, and pure metallic targets of Cu and Cr have all been explored for their unique advantages.

•For instance, CuCrO_2_ ceramic targets as stated by A. Barnabé et al., used in RF magnetron sputtering at a power of 150 W and a working pressure of 3.75 × 10^–3^ Torr, are known for producing films with excellent crystallinity and uniformity.^57^
•Oxide targets, such as Cu_2_O and Cr_2_O_3_ which have been discussed by Sundaresh et al., have been utilized lower power (50 W) and base pressure of (5 × 10^–7^ Torr), yielding films with high transparency and well-defined optoelectronic characteristics.^40^
•Cu:Cr with a ratio of 50:50 powder targets discussed by Yu R.-S. et al., employed at 200 W and a base pressure of 2 × 10^–6^ Torr, offer doping flexibility, enhancing the electrical properties of the films.^61^
•Pure metallic targets, such as Cu and Cr as stated in Bharath A. H. et al., are often used in DC magnetron sputtering at varied powers of both the targets (10–100 W) and working pressure (10 × 10^–3^ Torr), achieving films with excellent conductivity and stability.^41^

It is understood that there are several approaches to obtain in-phase CuCrO_2_. But based on the papers listed in Table 1, we conclude that a ceramic target for obtaining in-phase CuCrO_2_ is the most sought out method. The ceramic target has been used extensively so that in order to obtain the delafossite which is an single phase structure present among the many phases of these Copper oxides, it is much more convenient to obtain the single phase of CuCrO_2_ using the ceramic target with ease as we do not have to monitor the parameter for two different targets as seen in other methods.^84^ The usage of these ceramic targets also allows us to increase the electrical conductivity of this delafossite material by incorporating a dopant such as Mg.^42^ This has been done by optimizing the gas flow ratio,^58^ Sputtering power,^39^ and deposition/working pressure^85^ as shown in Figure 31.

•Sputtering power and pressure:

**Figure 31:**
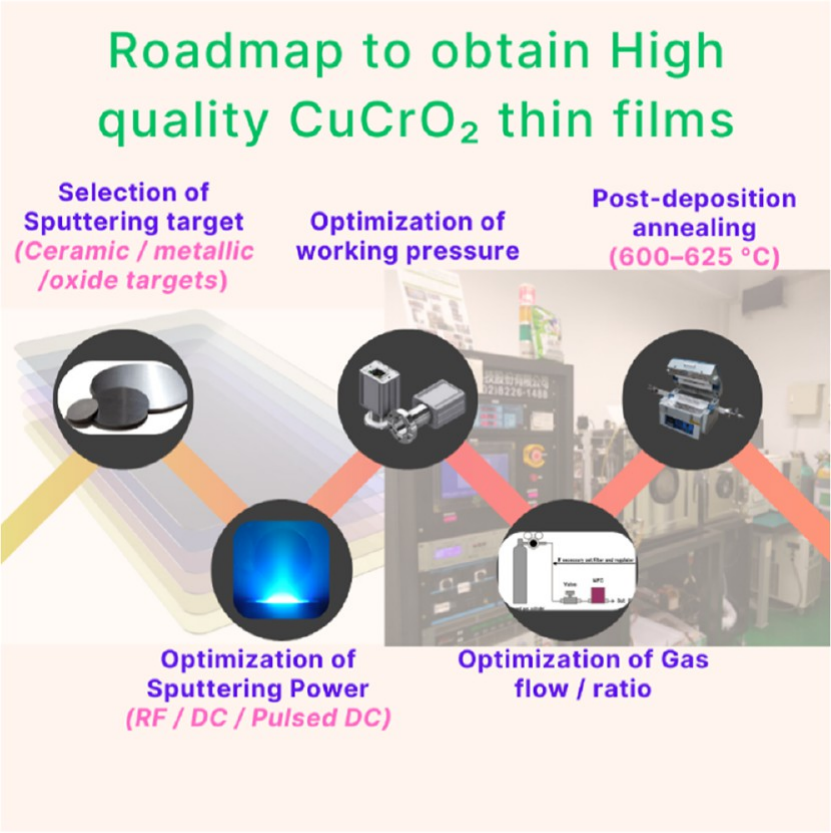
Roadmap for obtaining sputtered high-quality films of CuCrO_2_.

The sputtering power and chamber pressure are critical parameters in achieving phase-pure delafossite CuCrO_2_ crystals. Sputtering techniques, such as RF magnetron sputtering and DC magnetron sputtering, typically operate within power ranges of 50–200 W, enabling controlled thin-film deposition with thicknesses between 50 and 200 nm. The choice between RF and DC sputtering plays a crucial role in achieving high-quality, phase-pure CuCrO_2_ films, as each technique impacts deposition dynamics differently.

RF magnetron sputtering is generally preferred over DC magnetron sputtering for depositing CuCrO_2_ thin films due to its ability to sputter insulating and semiconducting targets efficiently. Since CuCrO_2_ has a relatively low electrical conductivity, DC sputtering can lead to charge buildup on the target surface, resulting in unstable plasma, nonuniform deposition, and poor film quality. In contrast, RF sputtering mitigates these issues by continuously alternating the electric field, allowing for a more stable discharge and improved film uniformity. Studies indicate that RF sputtering at lower power levels (e.g., ∼50 W) has successfully produced phase-pure CuCrO_2_, while higher power values in DC sputtering tend to promote secondary phases like CuO or Cr_2_O_3_. The necessity of optimizing the sputtering power is particularly evident in RF sputtering, where precise tuning ensures the formation of the delafossite phase without unwanted secondary phases. XRD and XPS characterizations confirm that RF magnetron sputtering at optimized power values results in superior crystallinity and phase purity compared with DC sputtering.

Therefore, RF magnetron sputtering is the preferred technique for obtaining high-quality CuCrO_2_ thin films, offering better plasma stability, enhanced phase purity, and improved film uniformity over DC sputtering, which is more suited for conducting materials. Irrespective of the sputtering technique and power settings, achieving high-quality delafossite CuCrO_2_ thin films requires careful optimization of multiple deposition parameters. Sputtering target composition, gas flow ratio, working pressure, and postdeposition treatment all play a crucial role in determining the phase purity of the material. Thus, it is essential for researchers to systematically optimize and understand the film formation dynamics to ensure successful growth of single-phase CuCrO_2_ thin films.

The working pressure during sputtering also plays a pivotal role in determining the quality and phase purity of CuCrO_2_ thin films. Typical working pressures range between 3 × 10^–3^ Torr and 5 × 10^–3^ Torr, providing a controlled environment that influences the kinetic energy of the sputtered particles and their subsequent deposition on the substrate. Low working pressures are particularly advantageous for achieving dense and uniform films with minimal defects as they allow sputtered species to maintain high energy, promoting effective crystallization. Furthermore, the interplay between the sputtering power and working pressure varies depending on the target material. For example, achieving higher film thicknesses for CuCrO_2_ often requires increased RF power in combination with lower working pressures,^62^ which enhances deposition rates without compromising film quality. These optimized conditions are essential for fabricating high-performance CuCrO_2_ thin films suitable for a range of applications, from optoelectronic devices to transparent conductive layers.

•Gas flow rate:

Gas ratios are another vital factor that significantly influences the sputtering process and the resulting film properties. The incorporation of gases such as Ar, N_2_, and O_2_ in specific ratios determines the chemical composition and phase purity of the deposited films. For instance, in RF magnetron sputtering with ceramic CuCrO_2_ targets, gas ratios of Ar/N_2_ at 1.0 Pa have been shown to enhance phase formation. Similarly, an Ar/O_2_ ratio of 30:8 sccm^62^ or an N_2_:Ar ratio of 40:60 is effective in producing doped delafossite films with minimal defects and improved optoelectronic performance such as higher electrical conductivity and lower sheet resistance as compared to the usage of the former gases such as Ar and O_2_ as the usage of N_2_. Optimized gas ratios are critical in achieving the perfect balance between crystallinity, conductivity, and transparency.

Conversely, increasing the O_2_/(O_2_+Ar) gas flow ratios during deposition and optimizing annealing temperatures contribute to enhanced transmittance in CuCrO_2_ thin films by improving phase purity and reducing defect density. The higher oxygen content facilitates stabilization of the Cu^1+^ oxidation state, crucial for the formation of the desired delafossite phase while minimizing the formation of unwanted secondary phases such as CuO and CuCr_2_O_4_. As a result, films exhibit better crystallinity, which enhances their optical properties. Additionally, studies have shown that the bandgap of CuCrO_2_ films tends to narrow as the film thickness increases due to increased carrier scattering. However, with improved crystallinity, the bandgap broadens, as reduced defect density leads to fewer scattering centers, ultimately contributing to better electrical conductivity and optoelectronic performance. Thus, optimizing both the gas flow ratios and annealing conditions is key to balancing the optical and electrical properties of CuCrO_2_ films for transparent electronic applications.

•Postdeposition Annealing:

Annealing plays a pivotal role in the deposition of CuCrO_2_ thin films by influencing the phase formation, crystallinity, and overall film quality. At intermediate temperatures (550–625 °C), annealing leads to the formation of secondary phases, such as CuO and CuCr_2_O_4_, producing polygonal and bar-like particle morphologies. While grain growth is initiated, phase segregation and incomplete crystallization can hinder the stability and purity of the CuCrO_2_ delafossite phase. However, when annealing is performed at higher temperatures (600–625 °C), a more stable CuCrO_2_ ^86^ phase is achieved, with improved crystallinity, larger grains, and reduced phase segregation. This temperature range fosters the growth of well-ordered grains, leading to a higher surface roughness and optimal film quality, which is essential for various optoelectronic applications.

Excessively high annealing temperatures (>700 °C)^87^ may introduce unwanted effects, such as atomic interdiffusion and the formation of rod-like Cr_2_O_3_ structures and voids near the substrate interface. This results in degradation of film stability and a trade-off between crystallinity and film integrity. Additionally, the optical properties of the films are closely linked to their structural characteristics, with increased thickness—often a result of higher sputtering power—leading to reduced optical transmittance due to enhanced light absorption and increased carrier density. Therefore, careful balance must be maintained between temperature, film thickness, and deposition parameters to achieve films with optimized electrical, optical, and structural properties. The annealing process is crucial for fine-tuning the material’s characteristics, ensuring the formation of high-quality CuCrO_2_ thin films suitable for transparent electronics and related applications.

Although the potential of low-temperature deposition of CuCrO_2_ thin films for flexible and wearable electronics is widely recognized, the majority of studies reported to date still rely on relatively high-temperature processes, typically exceeding 500 °C, to achieve phase-pure and highly crystalline films. Such high temperatures are necessary to promote atomic diffusion, facilitate grain growth, and stabilize the delafossite structure, thereby ensuring optimal optoelectronic properties such as high hole mobility and wide-bandgap transparency. However, these thermal requirements impose significant limitations on the integration of CuCrO_2_ with temperature-sensitive substrates, including polymers and other flexible materials commonly used in next-generation electronics. As a result, there is a notable absence of experimental studies that demonstrate the direct deposition and functional integration of CuCrO_2_ thin films onto flexible platforms while maintaining structural integrity, phase purity, and the desired electrical properties.

This gap highlights an important research opportunity: the development of deposition strategies and post-treatment protocols that allow for CuCrO_2_ crystallization at lower temperatures. Approaches such as plasma-assisted sputtering, chemical solution deposition with low-temperature annealing, ALD, or hybrid techniques combining mild thermal treatment with laser or microwave annealing could potentially overcome the current limitations. By enabling low-temperature growth, these strategies would facilitate the integration of CuCrO_2_ into flexible optoelectronic devices, including thin-film transistors, transparent electrodes, and wearable photovoltaics, while preserving mechanical flexibility and device performance.

Thus, while this review emphasizes established high-temperature deposition strategies for achieving phase-pure CuCrO_2_, it also underscores the critical need for research directed toward low-temperature processing, which remains an underexplored yet highly promising avenue for expanding the practical applications of delafossite thin films in flexible electronics.

In conclusion, obtaining perfect CuCrO_2_ phases requires a meticulous balance of deposition parameters, including the power source, target material, power value, operating pressure, and gas ratios. By optimizing these factors, high-quality CuCrO_2_ thin films with tailored properties can be achieved for various optoelectronic applications.

## 4 Delafossite CuCrO_2_ Thin Films for Device Applications

CuCrO_2_ thin films are widely utilized in various sensor applications due to their sensitivity to environmental changes. In gas sensors, CuCrO_2_ thin films detect gases such as CO, H_2_, and Volatile organic compounds by exhibiting changes in electrical conductivity proportional to the gas concentration.^88^ This characteristic makes them suitable for industrial settings, indoor air quality monitoring, and environmental monitoring, ensuring safety and reducing pollution. CuCrO_2_ thin films have demonstrated significant potential across a range of applications due to their unique properties. As environmental sensors, they effectively monitor air quality and pollution levels, enabling early warnings and protective measures.^82^ They also serve as chemical sensors, detecting and quantifying some chemicals in laboratory and industrial settings where precision is critical.^79^ CuCrO_2_’s sensitivity to temperature changes makes it suitable for use in temperature sensors and thermistors, vital for electronic and industrial process control. Additionally, these films are integrated into humidity sensors, measuring humidity levels by detecting changes in electrical properties, and pressure sensors, where they respond to pressure variations through changes in electrical conductivity.^89^ CuCrO_2_ thin films are also valuable in chemiresistive sensors for chemical analysis and safety, and in optical sensors, where their optical properties are utilized for measuring light intensity or wavelength in applications like optical communication and spectrometry.^90^

In biosensors, CuCrO_2_ films detect specific biological molecules or biomarkers, facilitating diagnostics and healthcare applications. They also serve as radiation sensors to measure ionizing radiation, crucial for radiation monitoring and safety. In thermoelectric applications, CuCrO_2_ converts waste heat into electricity, improving energy efficiency in industrial processes and automotive engines and providing power for portable electronics and wearable health monitoring devices.^45^ CuCrO_2_-based energy harvesters convert ambient energy sources, like temperature gradients or vibrations, into electrical energy for low-power electronics and sensors and are used in remote and off-grid power systems to store energy from intermittent sources like solar or wind.^79^ CuCrO_2_’s photodetection capabilities across various spectral ranges are also noteworthy. It is developed for visible light photodetectors used in ambient light sensing, optical communication, and image sensors. In spectroscopy, CuCrO_2_ photodetectors analyze material composition by measuring light intensity at different wavelengths, and they are used in environmental monitoring, smart lighting systems, and biomedical fluorescence imaging.^91^

While CuCrO_2_ is not commonly used in traditional solar cells, its wide bandgap and optical properties suggest potential for solar applications, though challenges remain in efficiency and stability. Significant research is needed to optimize its use in solar cell.^92^ CuCrO_2_ thin films, particularly those synthesized through sputtering, show great promise due to their high transparency, p-type conductivity, and wide bandgap. Sputtering allows precise control over film thickness and composition, resulting in uniform, defect-free films with excellent adherence to substrates.^85^ These films are ideal for optoelectronic devices, gas sensors, thermoelectric devices, and energy storage systems as described in Figure 32. The stability, reproducibility, and improved surface morphology achieved through sputtering enhance the performance of CuCrO_2_-based devices. Future research will focus on optimizing sputtering parameters to further improve film properties and expand application potential.^39^ Here are a few case studies showcasing the applications of CuCrO_2_ thin films.

**Figure 32:**
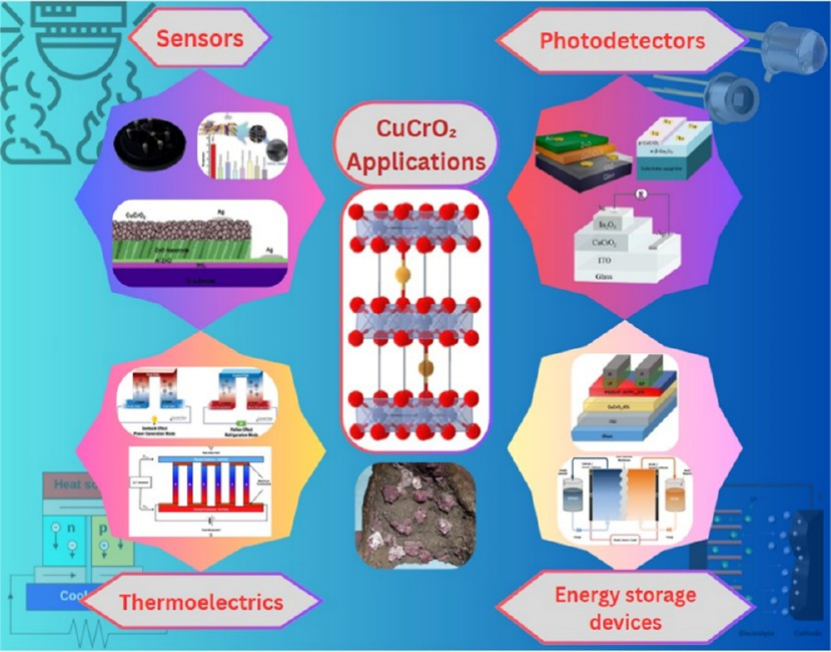
Diverse applications of CuCrO_2_ thin films reported in various domains.

A high-quality CuCrO_2_/ZnO heterojunction diode was successfully fabricated by Narro-Ríos, J. S. et al.^93^ using ultrasonic spray pyrolysis (shown in Figure 33a). The films exhibited a high density, as indicated by their refractive indices of 2.02 for ZnO and 1.97 for CuCrO_2_. I–V measurements revealed the diode’s characteristic rectification, with a performance figure approaching 10^7^ at ± 4.5 V, suggesting efficient charge carrier separation and transport within the device. Impedance spectroscopy identified four distinct activation energies corresponding to the bulk conductivities of CuCrO_2_ and ZnO and the interfaces between CuCrO_2_–ZnO and Au–CuCrO_2_. This detailed analysis underscores the complex interactions and conductive properties within the diode. The optical transmittance of the diode varied significantly, from 20% at 400 nm to 70% at 700 nm wavelengths, classifying it as a semitransparent diode. This variability in transmittance enables the device to be utilized in applications requiring both transparency and electrical functionality, making it a versatile component in optoelectronic applications.^94^

**Figure 33:**
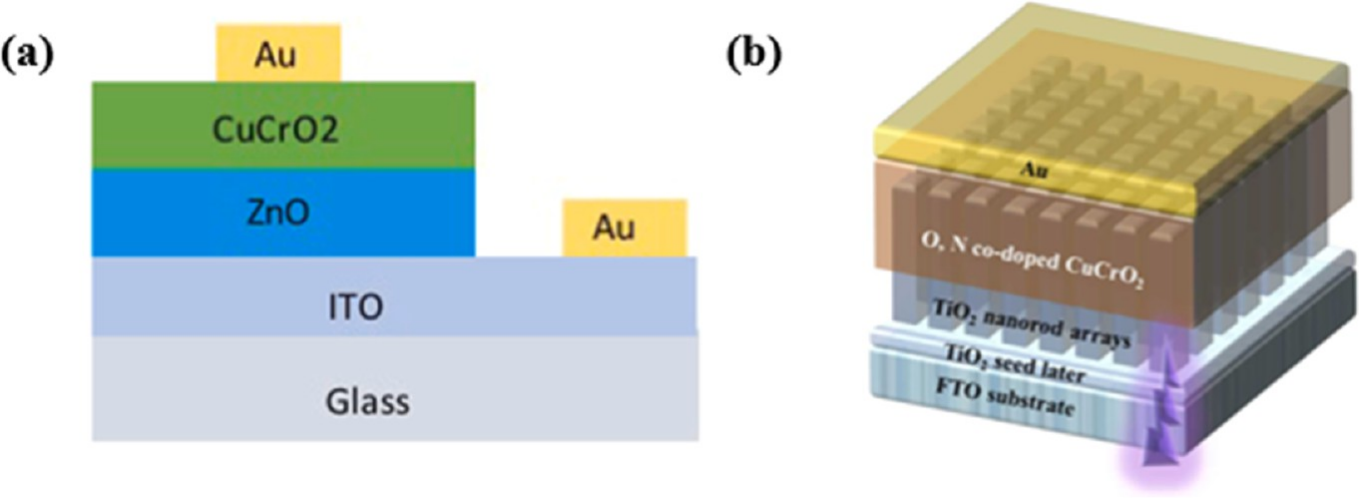
Schematic device structure of (a) CuCrO_2_/ZnO heterojunction diode. Reproduced with permission from ref 93. Copyright 2022, Elsevier. (b) UV photodetectors. Reproduced with permission from ref 91. Copyright 2024, Elsevier.

Huang, Meng, et al.^96^ optimized the optoelectronic properties and^95^ film-formation characteristics of CuCrO_2_ through oxygen (O) and nitrogen (N) codoping using the space-limited domain annealing method in ambient air (shown in Figure 33b). This codoping at 100 °C significantly enhanced the conductivity of CuCrO_2_ by decreasing trap-state density and increasing hole concentration, thereby facilitating carrier transport. With a work function of 5.24 eV and an electron affinity of 2.29 eV, the codoped CuCrO_2_ films function effectively as both a hole transport layer (HTL) and an electron-blocking layer. This dual role enhances the extraction of photogenerated holes, while suppressing nonradiative charge recombination at the heterojunction interface. As a result, the device with an FTO/TiO_2_ NRs/O, N-codoped CuCrO_2_/Au structure achieved a responsivity of 188 mA W^–1^ and a detectivity of 3.14 × 10^13^ cm Hz ^1/2^ W^–1^ in self-powered mode. Additionally, the device exhibited fast rise/decay times of 31.2/32.1 ns. Under a −2 V bias, the photodetector’s performance improved significantly, with a responsivity of up to 5 × 10^5^ mA/W (at 365 nm, 0.97 mW cm^–2^) and an apparent quantum efficiency of approximately 30,000%. This study underscores the potential of O, N-codoped CuCrO_2_ HTL in constructing high-performance optoelectronic devices.

Cossuet et al. fabricated an innovative self-powered UV photodetector using ZnO/CuCrO_2_ core–shell nanowire heterostructures (shown in Figure 34). This device was created with chemical deposition techniques at moderate temperatures. A uniform 35 nm-thick CuCrO_2_ shell, formed by aerosol-assisted chemical vapor deposition, envelops vertically aligned ZnO nanowires grown via chemical bath deposition. The heterostructures exhibit excellent diode behavior, with a rectification ratio of 1.2 × 10^4^ at ± 1 V and a high UV optical absorbance of over 85%. The photodetector demonstrated high UV responsivity at zero bias, reaching up to 3.43 mA W^–1^ under 365 nm UV light and 5.87 mA W^–1^ at 395 nm, with a UV-to-visible rejection ratio of 10^6^. The device also showed rapid response times, with rise and decay times of 32 and 35 μs, respectively, making it a promising candidate for cost-efficient, all-oxide self-powered UV photodetectors.^96^

**Figure 34:**
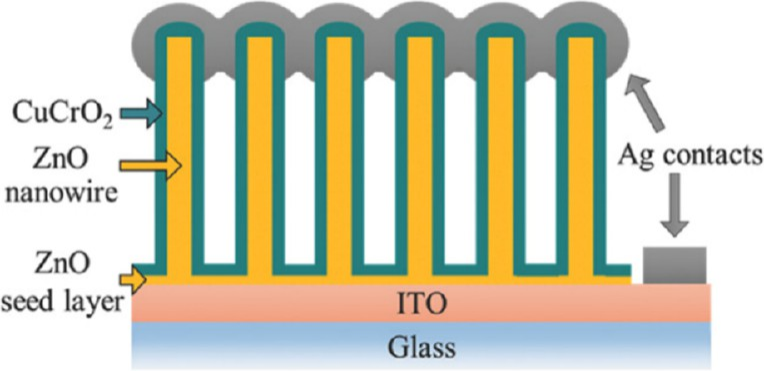
Schematic device structure of a self-powered UV photodetector. Reproduced with permission from ref 96 Copyright 2018, John Wiley and Sons.

In a study by Li, Yi et al., p-type delafossite CuCrO_2_ nanomaterials were synthesized via a one-step hydrothermal reaction (shown in Figure 35). These nanomaterials were used as the hole-transporting layer in an inverted stack organic photodiode (OPD). The organic photodiode exhibited a dark current density of 6.48 × 10^–8^ A cm^–2^ and an external quantum efficiency of 16.2% at 525 nm illumination under a −5 V bias. The device also demonstrated a responsivity of 68.5 mA W^–1^ and a detectivity of 4.75 × 10^11^ cm Hz^1/2^ W^–1^. Additionally, a large-area, flat-panel image sensor was developed using the same materials and structure applied onto a thin-film transistor backplane. This sensor featured an active area of 75.0 × 81.0 mm, a pitch of 150 μm, and a resolution of 510 × 470. The evaluation of the organic image sensor’s performance indicated that these materials and device configurations have significant potential for flat-panel detector applications.^96^

**Figure 35:**
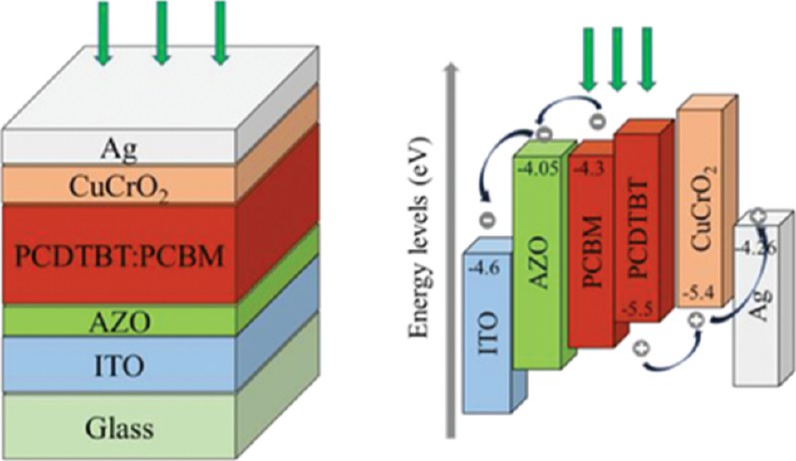
Schematic device structure of an inverted stack organic photodiode. Reproduced with permission from ref 96. Copyright 2021, John Wiley and Sons.

## 5 Conclusion and Future Prospects

This review provides a comprehensive analysis of sputtered CuCrO_2_ thin films, detailing their structural, morphological, optical, and electrical properties. The limited availability of high-performance p-type transparent conducting materials has long been a challenge in the development of advanced electronic and optoelectronic devices. CuCrO_2_ emerges as a promising candidate offering excellent optoelectrical characteristics. However, despite numerous studies, a lack of in-depth understanding regarding its characteristic properties and optimal deposition conditions has hindered its widespread application. Through this review, we have outlined and provided a roadmap to obtain the critical sputtering parameters and postdeposition treatments necessary to achieve high-quality, phase-pure CuCrO_2_ thin films. By addressing these optimization strategies, this study bridges the existing research gap and provides a quantitative analysis of the deposition conditions required for fabricating highly efficient thin films. Furthermore, the impact of doping has been explored, demonstrating how the material’s properties can be fine-tuned for specific applications. The versatility of CuCrO_2_ thin films has also been highlighted by discussing their potential applications in transparent electronics, photovoltaics, and optoelectronic devices. While significant progress has been made, several avenues remain open for future research:

•*Advanced Doping Strategies:* Further investigations into novel dopants and codoping techniques can enhance conductivity while maintaining high transparency.
•*Interface Engineering:* Understanding and optimizing heterostructure interfaces with other functional materials can expand the applicability of CuCrO_2_ thin films in next-generation devices.
•*Scalability and Industrial Integration:* Developing scalable sputtering techniques and integrating them into large-area deposition processes can facilitate commercialization.
•*Stability and Durability Studies:* Long-term environmental and thermal stability assessments are essential for practical device implementation.
•*Exploring Alternative Synthesis Routes:* While sputtering remains a preferred method, exploring hybrid deposition techniques or combinatorial approaches may unlock new possibilities for superior film quality.

In conclusion, the precise control of deposition techniques, strategic postprocessing modifications, and tailored doping approaches can significantly enhance the performance of CuCrO_2_ thin films. These advancements pave the way for next-generation optoelectronic and transparent electronic devices, marking a transformative step toward high-performance p-type transparent conducting materials.

## References

[1] King P. D. C., Veal T. D., McConville C. F., Zúñiga-Pérez J., Muñoz-Sanjosé V., Hopkinson M., Rienks E. D. L., Jensen M. F., Hofmann P. (2010). Surface Band-Gap Narrowing
in Quantized Electron Accumulation Layers. Phys.
Rev. Lett..

[2] Minami T. (2005). Transparent
Conducting Oxide Semiconductors for Transparent Electrodes. Semicond. Sci. Technol..

[3] Mudd J. J., Lee T. L., Muñoz-Sanjosé V., Zúñiga-Pérez J., Hesp D., Kahk J. M., Payne D. J., Egdell R. G., McConville C. F. (2014). Hard X-Ray
Photoelectron Spectroscopy as a Probe of the Intrinsic Electronic
Properties of CdO. Phys. Rev. B:Condens. Matter
Mater. Phys..

[4] Klein A., Körber C., Wachau A., Säuberlich F., Gassenbauer Y., Harvey S. P., Proffit D. E., Mason T. O. (2010). Transparent
Conducting Oxides for Photovoltaics: Manipulation of Fermi Level,
Work Function and Energy Band Alignment. Materials.

[5] Ellmer K. (2012). Past Achievements
and Future Challenges in the Development of Optically Transparent
Electrodes. Nat. Photonics.

[6] Yu X., Marks T. J., Facchetti A. (2016). Metal Oxides for Optoelectronic Applications. Nat. Mater..

[7] Zheng W. P., Rong Dai Z., Lin Wang Z. (1979). Nanobelts of Semiconducting Oxides. Science.

[8] Comini E., Faglia G., Sberveglieri G., Pan Z., Wang Z. L. (2002). Stable
and Highly Sensitive Gas Sensors Based on Semiconducting Oxide Nanobelts. Appl. Phys. Lett..

[9] Kawazoe H., Yanagi H., Ueda K., Hosono H. (2000). Transparent P-Type
Conducting Oxides: Design and Fabrication of p-n Heterojunctions. MRS Bull..

[10] Kawazoe H., Yasukawa M., Hyodo H., Kurita M., Yanagi H., Hosono H. P.-. (1997). P-type electrical conduction in transparent thin films
of CuAlO2. Nature.

[11] Hautier G., Miglio A., Ceder G., Rignanese G. M., Gonze X. (2013). Identification and Design Principles of Low Hole Effective Mass P-Type
Transparent Conducting Oxides. Nat. Commun..

[12] Nagarajan R., Draeseke A. D., Sleight A. W., Tate J. P.-. (2001). p-type conductivity
in CuCr1–xMgxO2 films and powders. J.
Appl. Phys..

[13] Bottiglieri L., Resende J., Weber M., Chaix-Pluchery O., Jiménez C., Deschanvres J. L. (2021). Out of Stoichiometry CuCrO2films
as a Promising P-Type TCO for Transparent Electronics. Mater. Adv..

[14] Jiang J., You Y. F., Vasu D., Chen S. C., Chiu T. W., Prashanth G., Chen P. C. (2023). Improving the P-Type CuCrO2 Thin
Film’s Electrical and Optical Properties. Materials.

[15] Shin D., Foord J. S., Egdell R. G., Walsh A. (2012). Electronic Structure
of CuCrO2 Thin Films Grown on Al 2O3(001) by Oxygen Plasma Assisted
Molecular Beam Epitaxy. J. Appl. Phys..

[16] Zhang N., Sun J., Gong H. (2019). Transparent P-Type Semiconductors: Copper-Based Oxides
and Oxychalcogenides. Coatings.

[17] Chown A. L., Farnum B. H. (2022). Defining the Role of Cr3+as a Reductant in the Hydrothermal
Synthesis of CuCrO2Delafossite. Inorg. Chem..

[18] Kim J., Kendall O., Ren J., Murdoch B. J., McConville C. F., van Embden J., Della Gaspera E. (2022). Highly Conductive and Visibly Transparent
P-Type CuCrO2 Films by Ultrasonic Spray Pyrolysis. ACS Appl. Mater. Interfaces.

[19] Baylan E., Akyildiz H., Yildirim O. A. (2019). Stable CuCrO2 Nanoparticles
- ZnO
Fibres p-n Heterostructure System for Effective Photocatalytic Activity. Process. Appl. Ceram..

[20] Chen H. Y., Wu J. T., Huang C. (2016). Development of a Fast Annealing Process
to Prepare Transparent Conductive Mg-Doped CuCrO2 Thin Films. Thin Solid Films.

[21] Mahapatra S., Shivashankar S. A., Wang X., Hsich M. T., Kim H. S., Gladfelter W. L., Yan J. H., Trans I., Wu H., Yang M. Y., Chin A., Chen W. J., Kwei C. M., Elec I., Osten H. J., Liu J. P., Gaworzewski P., Bugiel E., Zaumseil P., Im K., Yang H., Lee H., Sim H., Choi S., Jang T., Hwang H., Fissel A., Mussig H. J., Schwalke U., Boye K., Haberle K., Heller R., Hess G., Muller G., Ruland T., Tzscockel G., Phys Lett A., Reliab M., Williams A., Roberts J. L., Jones A. C., Chalker P. R., Tobin N. L., Bickley J. F., Davies H. O., Smith L. M., Leedham Chem Vap T. J., Crosbie M. J., Wright P. J., Steiner A., Leedham T. J., Critchlow G., Vap De C., Fleeting K. A., Otway D. J., Aspinall H. C., Williams P. A., Gaskell J., Critchlow G. W., Lane P. A., Williams D. J., Suddhasatta Mahapatra B., Shivashankar S. A. (2003). Low-Pressure Metal–Organic Chemical Vapor Deposition
of Transparent and p-Type Conducting CuCrO2 Thin Films with High Conductivity. Chem. Vap. Deposition.

[22] Li D., Fang X., Deng Z., Zhou S., Tao R., Dong W., Wang T., Zhao Y., Meng G., Zhu X. (2007). Electrical, Optical and Structural Properties of CuCrO2 Films Prepared
by Pulsed Laser Deposition. J. Phys. D Appl.
Phys..

[23] Tripathi T. S., Karppinen M. (2015). Structural
Optical and Electrical Transport Properties
of ALD-Fabricated CuCrO2 Films. Phys. Procedia.

[24] Oechsner H. (1975). Sputtering-a
Review of Some Recent Experimental and Theoretical Aspects. Appl. Phys..

[25] Lehmann C., Sigmund P. (1966). On the Mechanism of Sputtering. Phys. Status Solidi B.

[26] Sarakinos K., Alami J., Konstantinidis S. (2010). High Power
Pulsed Magnetron Sputtering:
A Review on Scientific and Engineering State of the Art. Surf. Coat. Technol..

[27] Sun H., Arab Pour
Yazdi M., Briois P., Pierson J. F., Sanchette F., Billard A. (2015). Towards Delafossite Structure of Cu–Cr–O
Thin Films Deposited by Reactive Magnetron Sputtering: Influence of
Substrate Temperature on Optoelectronics Properties. Vacuum.

[28] Dong G., Zhang M., Zhao X., Yan H., Tian C., Ren Y. (2010). Improving the Electrical Conductivity
of CuCrO2 Thin Film by N Doping. Appl. Surf.
Sci..

[29] Barnabé A., Thimont Y., Lalanne M., Presmanes L., Tailhades P. (2015). P-Type Conducting Transparent Characteristics of Delafossite
Mg-Doped CuCrO 2 Thin Films Prepared by RF-Sputtering. J. Mater. Chem. C Mater..

[30] Chidambaram D., Clayton C. R., Halada G. P., Jung M.-H., Choi H.-S., Ralston K. D., Young T. L. (2008). The Optical and Electrical Properties
of Zn-Doped CuAlO2 Thin Films Deposited by RF Magnetron Sputtering. J. Electrochem. Soc..

[31] Chaipech K., Kamwanna T., Phokha S., Yensano R. (2024). Structural and Room
Temperature Ferromagnetic Properties of Fe3+-Doped CuCrO2 Fibers. Mater. Chem. Phys..

[32] Jamshina
Sanam P. K., Shah M., Pradyumnan P. P. (2023). Magnetoelectric
Coupling and Thermoelectric Behaviors in Ni-Doped CuCrO2 Crystallites. Chem. Eng. J..

[33] Bharath S. P., Kumar A., Kumar M. (2025). Impedance-Based
Multivariate Analysis
for Accurate Estimation of H2S Concentrations Using CuCrO2 Gas Sensor. Sens. Actuators, B.

[34] Ratz T., Fourneau E., Sliti N., Malherbe C., Baret A., Vertruyen B., Silhanek A. V., Nguyen N. D. (2025). Correlation between
Material Properties, Crystalline Transitions, and Point Defects in
RF Sputtered (N,Mg)-Doped Copper Oxide Thin Films. ACS Appl. Electron. Mater..

[35] Ma X., Ren P., Zhang S., He X., Li Y., Yin X., Li H., Dang S., Yu D., Qiu J., Zhou X., Zhou B. (2025). A Significant Improvement in Corrosion Resistance and Biocompatibility
in ZrNbTiCrCu High-Entropy Films Induced by the Precipitation of Cu. J. Mater. Sci. Technol..

[36] Bharath A. H., Sundaram K. B. (2024). Characterization
of Reactively Sputter Deposited CuCrO
2 Thin Films Using Cu and Cr Targets. Mater.
Adv..

[37] Jamshina
Sanam P. K., Shah M., Pradyumnan P. P. (2024). Tailoring
Structure and Nanoscale Surface Topography in Mg–N Doped CuCrO2
Thin Films via Post Deposition Annealing for Optothermoelectric Application. Opt Mater..

[38] Tsai D.-C., Chen E.-C., Huang Y.-L., Shieu F.-S., Chang Z.-C. (2023). Annealing
Effect on the Structural and Optoelectronic Properties of Cu-Cr-O
Thin Films Deposited by Reactive Magnetron Sputtering Using a Single
CuCr Target. Mater. Sci.-Pol..

[39] Van
Hoang D., Duc Thi Dinh H., Huu Nguyen T., Tuan Thanh Pham A., Doan Thi T. U., Thuy Thi Phan T., Huu Nguyen K., Bach Phan T., Cao Tran V. (2023). Fine-Tuning High Electrical
Conductivity of Mg-Doped CuCrO2 Delafossite Thin Films through Preferred-(110)
Orientation and Film Thickness Control. Phys.
B.

[40] Sundaresh S., Bharath A. H., Sundaram K. B. (2023). Effect
of Annealing Temperature on
Radio Frequency Sputtered P-Type Delafossite Copper Chromium Oxide
(CuCrO2) Thin Films and Investigation of Diode Characteristics Forming
Transparent Pn-Heterojunction. Coatings.

[41] Sundaresh S., Bharath A. H., Sundaram K. B. (2023). Effect of Cu2O Sputtering
Power Variation
on the Characteristics of Radio Frequency Sputtered P-Type Delafossite
CuCrO2 Thin Films. Coatings.

[42] Van
Hoang D., Tuan Thanh Pham A., Baba T., Huu Nguyen T., Bao Nguyen Le T., Dieu Thi Ung T., Hong J., Bae J. S., Park H., Park S., Ohkubo I., Mori T., Cao Tran V., Bach Phan T. (2022). New Record High Thermoelectric ZT
of Delafossite-Based CuCrO2 Thin Films Obtained by Simultaneously
Reducing Electrical Resistivity and Thermal Conductivity via Heavy
Doping with Controlled Residual Stress. Appl.
Surf. Sci..

[43] Ahmadi M., Abrari M., Ghanaatshoar M. (2021). An All-Sputtered
Photovoltaic Ultraviolet
Photodetector Based on Co-Doped CuCrO2 and Al-Doped ZnO Heterojunction. Sci. Rep..

[44] Okada T., Usui S., Kawashima T., Washio K. (2021). Investigation of Crystallinity,
Electrical Conductivity, and Optical Transmittance of Mg-Doped CuCrO2
Deposited on Buffer Layer. Mater. Sci. Semicond.
Process..

[45] Ahmadi M., Anaghizi S. J., Asemi M., Ghanaatshoar M. (2021). Plasma-Treated
Room Temperature Synthesized CuCrO2/Au/CuCrO2 on Polyethylene Terephthalate:
Towards a High-Performance Flexible p-Type Transparent Conductor. Thin Solid Films.

[46] Ohno K., Okada T., Kawashima T., Washio K. (2020). Effect of Forming Gas
Annealing on Improvement in Crystal Orientation of Solid-Phase Calcined
CuCrO2 Thin Film. Thin Solid Films.

[47] Lin S. S., Shi Q., Dai M. J., Wang K. L., Chen S. C., Kuo T. Y., Liu D. G., Song S. M., Sun H. (2020). The Optoelectronic
Properties of P-Type Cr-Deficient Cu­[Cr0.95–xMg0.05]­O2 Films
Deposited by Reactive Magnetron Sputtering. Materials.

[48] Tsai D. C., Chang Z. C., Kuo B. H., Chen C. M., Chen E. C., Shieu F. S. (2019). Influence of Chemical
Composition on Phase Transformation
and Optoelectronic Properties of Cu–Cr–O Thin Films
by Reactive Magnetron Sputtering. J. Mater.
Res. Technol..

[49] Ahmadi M., Asemi M., Ghanaatshoar M. (2018). Improving
the Electrical and Optical
Properties of CuCrO2 Thin Film Deposited by Reactive RF Magnetron
Sputtering in Controlled N2/Ar Atmosphere. Appl.
Phys. A Mater. Sci. Process.

[50] Chiba H., Hosaka N., Kawashima T., Washio K. (2018). Thermal Solid-Phase
Crystallization of Amorphous CuCrO2:N Thin Films Deposited by Reactive
Radio-Frequency Magnetron Sputtering. Thin Solid
Films.

[51] Sinnarasa I., Thimont Y., Presmanes L., Bonningue C., Barnabé A., Tailhades P. (2018). Influence
of Thickness and Microstructure
on Thermoelectric Properties of Mg-Doped CuCrO2 Delafossite Thin Films
Deposited by RF-Magnetron Sputtering. Appl.
Surf. Sci..

[52] Chiba H., Kawashima T., Washio K. (2017). Optical and Structural Properties
of CuCrO2 Thin Films on C-Face Sapphire Substrate Deposited by Reactive
RF Magnetron Sputtering. Mater. Sci. Semicond.
Process..

[53] Sun H., Arab Pour Yazdi M., Ducros C., Chen S. C., Aubry E., Wen C. K., Hsieh J. H., Sanchette F., Billard A. (2017). Thickness-Dependent
Optoelectronic Properties of CuCr0.93Mg0.07O2
Thin Films Deposited by Reactive Magnetron Sputtering. Mater. Sci. Semicond. Process..

[54] Sinnarasa I., Thimont Y., Presmanes L., Barnabé A., Tailhades P. (2017). Thermoelectric and Transport Properties
of Delafossite
CuCrO2:Mg Thin Films Prepared by RF Magnetron Sputtering. Nanomaterials.

[55] Sun C. H., Tsai D. C., Chang Z. C., Chen E. C., Shieu F. S. (2016). Effects
of Annealing Time on the Structural and Optoelectronic Properties
of P-Type Conductive Transparent Cu–Cr–O Films. J. Mater. Sci.: Mater. Electron..

[56] Yu R.-S., Wang M.-C. (2016). Plasma Annealing
Effects on the Material Characteristics
of Sputtering Deposited CuCrO 2 Thin Films. ECS J. Solid State Sci. Technol..

[57] Barnabé A., Thimont Y., Lalanne M., Presmanes L., Tailhades P. P.-. (2015). p-Type conducting transparent characteristics of delafossite
Mg-doped CuCrO_2_ thin films prepared by RF-sputtering. J. Mater. Chem. C Mater..

[58] Wu S., Deng Z., Dong W., Shao J., Fang X. (2015). Effect of
Deposition Atmosphere on the Structure and Properties of Mg Doped
CuCrO2 Thin Films Prepared by Direct Current Magnetron Sputtering. Thin Solid Films.

[59] Yu R. S., Tasi C. P. (2014). Structure, Composition
and Properties of p-Type CuCrO2
Thin Films. Ceram. Int..

[60] Wu S.Z., Deng Z.H., Dong W.W., Shao J.Z., Fang X.D. (2014). Deposition
and Characterization of Mg Doped CuCrO_2_ Films by DC Magnetron
Sputtering. Key Eng. Mater..

[61] Yu R. S., Wu C. M. (2013). Characteristics
of P-Type Transparent Conductive CuCrO2 Thin Films. Appl. Surf. Sci..

[62] Chiu T. W., Yang Y. C., Yeh A. C., Wang Y. P., Feng Y. W. (2013). Antibacterial
Property of CuCrO2 Thin Films Prepared by RF Magnetron Sputtering
Deposition. Vacuum.

[63] Cullity, B. D. ; Bernard, D. Elements of X-Ray Diffraction; Addison-Wesley Publishing Company, Inc, 1978.

[64] Kudelski A. (2008). Analytical
Applications of Raman Spectroscopy. Talanta.

[65] Van
Hoang D., Duc Thi Dinh H., Huu Nguyen T., Tuan Thanh Pham A., Doan Thi T. U., Thuy Thi Phan T., Huu Nguyen K., Bach Phan T., Cao Tran V. (2023). Fine-Tuning High Electrical
Conductivity of Mg-Doped CuCrO2 Delafossite Thin Films through Preferred-(110)
Orientation and Film Thickness Control. Phys.
B.

[66] Garg A. B., Rao R. (2018). Copper Delafossites
under High Pressure—A Brief Review of
XRD and Raman Spectroscopic Studies. Crystals.

[67] Jamshina
Sanam P. K., Shah M., Pradyumnan P. P. (2023). The Enhancement
of NIR Transparency Due to Annealing and Mg-Doping in CuCrO2 Thin
Films. Mater. Lett..

[68] Chen C. Y., Sakthinathan S., Yu C. L., Wang C. C., Chiu T. W., Han Q. (2021). Preparation and Characterization of Delafossite CuCrO2 Film on Flexible
Substrate. Ceram. Int..

[69] Andrade, J. D. X-Ray Photoelectron Spectroscopy (XPS). Surface and Interfacial Aspects of Biomedical Polymers; Springer US, 1985, pp 105–195.

[70] John L., Mrinaleni R. S., Amaladass E. P., Pan S., Prabhu E., Sivaraman N., Gnanasekar K. I. (2024). Electrical Conductivity, Carrier
Concentration, Mobility and XPS Studies on Thin Films of Metallic
PdCoO2 Delafossite. Appl. Phys. A Mater. Sci.
Process.

[71] Akash, M. S. H. ; Rehman, K. Essentials of Pharmaceutical Analysis; Springer Singapore, 2019.

[72] UV–vis and Photoluminescence Spectroscopy for Nanomaterials Characterization.

[73] Ponmudi S., Sivakumar R., Sanjeeviraja C. (2023). Tuning the Phase Structure and Surface
Morphology of Cr2O3:CuO Thin Film by Annealing for Enhanced Ammonia
Sensing Performance at Room Temperature. Inorg.
Chem. Commun..

[74] Nicolas, B. ; Hendrix, D. ; Raynald, G. Field Emission Scanning Electron Microscopy: New Perspectives for Materials; Springer, 2017, pp 145–159.

[75] Tang C. Y., Yang Z. (2017). Transmission Electron Microscopy (TEM). Membrane
Characterization.

[76] Mohammed, A. ; Abdullah, A. SCANNING ELECTRON MICROSCOPY (SEM). Proceedings of 2018 International Conference on Hydraulics and Pneumatics-HERVEX.

[77] Voigtländer, B. NanoScience and Technology Atomic Force Microscopy, 2nd ed.; Springer Cham, 2019.

[78] Tripathi T.
S., Niemelä J. P., Karppinen M. (2015). Atomic Layer Deposition of Transparent
Semiconducting Oxide CuCrO 2 Thin Films. J.
Mater. Chem. C Mater..

[79] Liu H., Cao X., Wu H., Li B., Li Y., Zhu W., Yang Z., Huang Y. (2020). Innovative Development on a P-Type
Delafossite CuCrO2 Nanoparticles Based Triethylamine Sensor. Sens. Actuators, B.

[80] Sanam P. K. J., Shah M., Pradyumnan P. P. (2022). Raman Spectroscopic
Investigation
and Thermoelectric Studies of Defect-Induced Mg-Doped Delafossite
Thin Film. J. Mater. Sci.: Mater. Electron..

[81] Yu R. S., Chu C. (2019). Synthesis and Characteristics
of Zn-Doped CuCrO2 Transparent Conductive
Thin Films. Coatings.

[82] Li R., Xu W., Lu X., Fang Y., Xu M., Luo C., Peng H., Lin H., Duan C. (2023). Dual Selective Gas
Sensor Based on Delafossite CuCoO2 with Instantaneously Attenuated
Response to Amine at Room Temperature. ACS Appl.
Electron. Mater..

[83] Liu H., Cao X., Wu H., Li B., Li Y., Zhu W., Yang Z., Huang Y. (2020). Innovative Development on a P-Type
Delafossite CuCrO2 Nanoparticles Based Triethylamine Sensor. Sens. Actuators, B.

[84] Veron F., Pasquet I., Thimont Y., Barnabé A., Tailhades P. (2022). Improved Performance of Transparent
P-Type Conductors
CuCrO2:Mg Delafossite Thin Films through Easy and Low Cost Laser Annealing. Mater. Lett..

[85] Sarkar D. K., Mahmud Hasan A. K., Mottakin M., Selvanathan V., Sobayel K., Ariful Islam M., Muhammad G., Aminuzzaman M., Shahiduzzaman M., Sopian K., Akhtaruzzaman M. (2022). Lead Free
Efficient Perovskite Solar Cell Device Optimization and Defect Study
Using Mg Doped CuCrO2 as HTL and WO3 as ETL. Sol. Energy.

[86] Chen H.-Y., Chang K.-P. (2013). Influence of Post-Annealing
Conditions on the Formation
of Delafossite-CuCrO 2 Films. ECS J. Solid State
Sci. Technol..

[87] Chen H.-Y., Chang K.-P. (2013). Influence of Post-Annealing
Conditions on the Formation
of Delafossite-CuCrO 2 Films. ECS J. Solid State
Sci. Technol..

[88] Xu S., Zhao T., Kong L., Zhu W., Bo M., Huang Y., Liu H. (2021). Gas-Solid Interfacial
Charge Transfer
in Volatile Organic Compound Detection by CuCrO2 Nanoparticles. Nanotechnology.

[89] Sakthinathan S., Rajakumaran R., Keyan A. K., Yu C. L., Wu C. F., Vinothini S., Chen S. M., Chiu T. W. (2021). Novel Construction
of Carbon Nanofiber/CuCrO2composite for Selective Determination of
4-Nitrophenol in Environmental Samples and for Supercapacitor Application. RSC Adv..

[90] Lei H. J., Su H. M., Vasu D., You Y. F., Chiu T. W., Vittayakorn N. (2023). Highly Efficient CeO2–CuCrO2 Composite Nanofibers
Used for Electrochemical Detection of Dopamine in Biomedical Applications. Fibers.

[91] Huang M., Wang Y., Zhang H., Mao M., Cen B., Wang T., Zhang Z., Li Q., Liu K., Kong P., Zhang J., Luo S., Luo G. O. (2024). N Co-Doped
CuCrO2 as Efficient Hole Transport Layer for High-Performance Ultraviolet
Photodetectors. J. Alloys Compd..

[92] Zhang H., Wang H., Zhu H., Chueh C. C., Chen W., Yang S., Jen A. K. Y. (2018). Low-Temperature
Solution-Processed
CuCrO2 Hole-Transporting Layer for Efficient and Photostable Perovskite
Solar Cells. Adv. Energy Mater..

[93] Narro-Ríos J. S., Garduño-Wilches I., Alarcón-Flores G., Ruiz-Rojas C. A., Gómez-Lizárraga K., Aguilar-Frutis M. (2022). Spray Pyrolysis Synthesis of a Semi-Transparent p-CuCrO2/n-ZnO
Heterojunction: Structural, Optical, and Electrical Properties. Phys. B.

[94] Liu A., Zhu H., Kim M. G., Kim J., Noh Y. Y. (2021). Engineering Copper
Iodide (CuI) for Multifunctional p-Type Transparent Semiconductors
and Conductors. Adv. Sci..

[95] Cossuet T., Resende J., Rapenne L., Chaix-Pluchery O., Jiménez C., Renou G., Pearson A. J., Hoye R. L. Z., Blanc-Pelissier D., Nguyen N. D., Appert E., Muñoz-Rojas D., Consonni V., Deschanvres J. L. (2018). ZnO/CuCrO2
Core–Shell Nanowire Heterostructures for Self-Powered UV Photodetectors
with Fast Response. Adv. Funct. Mater..

[96] Li Y., Luo H., Mao L., Yu L., Li X., Jin L., Zhang J. (2021). A Solution-Processed Hole-Transporting Layer Based
on p-Type CuCrO2
for Organic Photodetector and Image Sensor. Adv. Mater. Interfaces.

